# Comprehensive assessment of microalgal-based treatment processes for dairy wastewater

**DOI:** 10.3389/fbioe.2024.1425933

**Published:** 2024-08-06

**Authors:** Pooja Singh, Satya Sundar Mohanty, Kaustubha Mohanty

**Affiliations:** ^1^ Department of Chemical Engineering, Indian Institute of Technology Guwahati, Guwahati, India; ^2^ Division of Biotechnology, Karunya Institute of Technology and Sciences, Coimbatore, India

**Keywords:** biofuel, bio-products, dairy wastewater, microalgae, nutrient removal, photobioreactors

## Abstract

The dairy industry is becoming one of the biggest sectors within the global food industry, and these industries use almost 34% of the water. The amount of water used is governed by the production process and the technologies employed in the plants. Consequently, the dairy industries generate almost 0.2–10 L of wastewater per liter of processed milk, which must be treated before being discharged into water bodies. The cultivation of microalgae in a mixotrophic regime using dairy wastewater enhances biomass growth, productivity, and the accumulation of value-added product. The generated biomass can be converted into biofuels, thus limiting the dependence on petroleum-based crude oil. To fulfill the algal biorefinery model, it is important to utilize every waste stream in a cascade loop. Additionally, the harvested water generated from algal biomass production can be recycled for further microalgal growth. Economic and sustainable wastewater management, along with proper reclamation of nutrients from dairy wastewater, is a promising approach to mitigate the problem of water scarcity. A bibliometric study revealing limited work on dairy wastewater treatment using microalgae for biofuel production. And, limited work is reported on the pretreatment of dairy wastewater via physicochemical methods before microalgal-based treatment. There are still significant gaps remains in large-scale cultivation processes. It is also crucial to discover robust strains that are highly compatible with the specific concentration of contaminants, as this will lead to increased yields and productivity for the targeted bio-product. Finally, research on reutilization of culture media in photobioreactor is necessary to augument the productivity of the entire process. Therefore, the incorporation of the microalgal biorefinery with the wastewater treatment concept has great potential for promoting ecological sustainability.

## 1 Introduction

The exhaustion of fossil fuels in transportation and industrial sectors has resulted in the depletion of already available natural resources together with the emission of notable greenhouse gases. Nowadays, studies are focused on finding renewable energy feedstock to overcome the coming energy crisis and to lower down the footprint of global warming (Chandra et al., 2021). Microalgae are gaining global recognition for their potential uses in several industries, including bioenergy, medicines, aquaculture, food, agriculture, and bioplastics (Arora et al., 2021; Agarwalla et al., 2023; Ramírez Mérida and Rodríguez Padrón, 2023; Agarwalla and Mohanty, 2024). Furthermore, there is research going on the potential of microalgae to remove calcitrant and harmful compounds, with the aim of industrial wastewater treatment. Industrial-scale growth of microalgae necessitates the use of large quantities of water and nutrients. Utilizing nonpotable water for growing microalgae may reduce the need for freshwater. The lower biomass productivity and elevated expense of growth media are the challenges linked with the production of microalgal biomass for various applications. The utilization of industrial effluent for cultivating microalgae has become a substitute to decrease the costs of the process and to produce biomass. The dairy industries are operating worldwide, but the cost of manufacturing processes differs from area to area. India, with a large population dependent on livestock and agriculture for their income, has emerged as a significant hub for the dairy industry. Almost 200–10,000 mL of wastewater is produced/L of processed milk and it must be treated before discharging to water bodies (Chandra et al., 2021). The effluent generated from the dairy industry has a high concentration of organic compounds and is not considered safe due to its high chemical oxygen demand (COD) and biochemical oxygen demand (BOD). Nevertheless, this dairy wastewater (DW) could be an excellent source of nutrients for microalgae which is rich in sugars, amino acids, ammonium, and phosphates sources (Vieira Costa et al., 2021). Cultivating the microalgae in dairy effluents has numerous benefits, including the ability to grow using available nutrients in the wastewater without adding additional nutrients (Singh et al., 2023; Ravi Kiran et al., 2024). This process also reduces the COD and BOD of the effluent, providing a cost-effective method for treating dairy waste. Additionally, it offers the potential to extract valuable products like lipids, proteins, and carbohydrates for various applications (Chandra et al., 2021). This review investigation aims to present recent studies in the area of microalgal-based dairy wastewater treatment. This is the first study to present a scientometric analysis of dairy wastewater treatment using microalgae. Also, a comprehensive assessment was conducted on previous studies in this review literature. In this literature, the importance of cultivation of microalgae in dairy effluent was highlighted as a source of growth media which can reduce the cost of the overall biomass generation process. Additionally, this review presents the characteristics of the different dairy wastewater and the efficiency of microalgae in the remediation of wastewater.

### 1.1 Background and significance of dairy wastewater

Due to the increased public awareness regarding nutrition and health over the past 45 years, the dairy industry has developed into a highly organized sector. There are over 101,000 number of milk co-operatives operating throughout the nation to provide milk processing, distribution, and large-scale production through a large number of dairies. With a milk production of 155.2 million tonnes in 2016–17, India has surpassed all other countries to take the lead globally. The cooperative/government and private sectors collectively operate over a thousand dairies that produce an estimated 100 million liters of milk daily (Singh et al., 2023). Within the global food industry, the dairy market is one of the biggest sectors. The dairy farmers in the food industry use 34% of the water. The amount of water used is governed by the production process and the technologies used in the plant. The application of sufficient hygienic levels in manufacturing and the urge to decrease the usage of water and enhance the efficacy of the treatment of wastewater are challenges that demand different approaches. The dairy sector consumes between 1,000 and 10,000 L of water for processing 1,000 L of milk. Based on data from the FAO (Food and Agriculture Organisation), global milk production has seen a significant growth of over 59% in the past 30 years. Specifically, it has risen from 530 million tonnes in 1988 to 843 million tonnes in 2018. With a quarter of the world’s milk produced, India leads the world’s milk producers, followed by the US, China, Pakistan, and Brazil. DW refers to the effluent that is produced by dairy organizations (Aziz and Ali, 2017). It has a whitish shade, accompanied by an unpleasant odor and a cloudy appearance (Kolev Slavov, 2017). Dairy wastewater includes significant amounts of milk solids, fats, nutrients, lactose, detergents, and sterilizing agents which correspond to elevated levels of biological oxygen demand ranging from 40 to 48,000 mg L^-1^, chemical oxygen demand ranging from 80 to 95,000 mg L^-1^, and pH values that vary between 4 and 11. The difference in pH levels is attributed to the presence of different detergents in the water for cleaning (Vieira Costa et al., 2021). The inappropriate release of DW into water bodies without adequate treatment not only leads to environmental contamination but also impacts nearby groundwater and reservoirs of water, thus adversely affecting human health (Garcha et al., 2016). This phenomenon arises from the rapid degradation of DW components. As a result, the concentrations of dissolved oxygen in the water bodies are depleted that receive these effluents. Consequently, these streams become breeding grounds for disease-carrying insects (Vieira Costa et al., 2021). Also, these industries generate effluents that include high concentrations of oils and greases, which have detrimental effects on wastewater treatment systems. The oil and grease content of raw dairy wastewater collected from an unorganized dairy industry of Patiala, Ludhiana, Shri Muktsar Sahib, and Bathinda (Punjab, India) was found to be within the range of 218–700 mg L^-1^ (Garcha et al., 2016). These effluents often lead to foul odors and obstructions in pipes. The wastewater also includes significant levels of nutrients, which may support the growth of several unidentified bacteria. Therefore, to accomplish effective biological remediation, it is crucial to ascertain the bacterial composition in the wastewater (Vieira Costa et al., 2021). Dairy wastewater often includes a significant abundance of heterotrophic bacteria, including species such as *Pseudomonas* (Alalam et al., 2021), *Bacillus cereus* (Garcha et al., 2016), *Enterobacter* (Alalam et al., 2021), *Streptococcus* (Alalam et al., 2021), and *Escherichia coli* (Boutilier et al., 2009). Microalgae can efficaciously utilize the nutrients available in DW and simultaneously produce valuable products. The amalgamation of algae cultivation with dairy wastewater treatment yields significant advantages, including the conservation of water resources, cost-effective bioremediation of the wastewater, generation of biomass suitable for bioenergy and animal feed, and the emergence of possibilities for the development of other high-value products (Singh et al., 2023).

### 1.2 Importance of microalgal-based treatment processes

Microalgae are photosynthetic organisms, may exist as single-celled or multi-celled organisms, and survive in both freshwater and saltwater ecosystems. These can effectively use carbon dioxide, light, and water to produce a range of valuable bioactive chemicals, including carbohydrates, proteins, and lipids. Various studies utilize microalgal biomass for other commercially valuable purposes. These include extracting pigments and vitamins for animal feed (Vieira Costa et al., 2021), and producing antioxidant, antitumor, anti-inflammatory, and antimicrobial compounds (Divya Kuravi and Venkata Mohan, 2021; Kiran and Venkata Mohan, 2022), generating biofuels (Singh et al., 2023; Ravi Kiran et al., 2024). Microalgae have been extensively employed for wastewater treatment. The two genera being the most extensively cultivated and studied worldwide in recent times were *Chlorella* and *Spirulina* (Vieira Costa et al., 2021). The process of phycoremediation involves first selecting the species and mode of cultivation, followed by pretreatment for product extraction and purification. However, the cost of cell productivity, contamination, and low yield are challenges that must be overcome to enable the scalability of this process (Vieira Costa et al., 2021). The primary physicochemical parameters that influence cell productivity are light intensity, nutrient, pH value, temperature, CO_2_ amount, salinity, and aeration. The light intensity and concentrations of nutrients are limiting variables for the growth of microalgal cells during the cultivation period. The intensity and length of light throughout the photoperiod control the photosynthesis energy supply, whereas the nutrient content directly affects the cellular metabolism and structure. Carbon, phosphate, and nitrogen are regarded as the most vital components of the metabolic pathway for photosynthesis. The metal ions present in the wastewater help in osmoregulation and the molecular configuration of photosynthetic complexes. The most critical parameter to consider when cultivating the microalgae is the pH. Maintaining the pH within the optimal range for microalgal cultivation promotes biomass production, which is also an important factor in terms of the solubility of nutrients (Vieira Costa et al., 2021). These conditions account for 30% of production costs in large-scale microalgae production. The bioreactor design and harvesting method are also a crucial factors in influencing the efficiency of large-scale biomass generation and overall production cost (Agarwalla et al., 2023; Agarwalla and Mohanty, 2024).

### 1.3 Importance of clean water generation and high-value product extraction

Post-harvesting represents an additional significant obstacle in the generation of microalgae biomass. Typically, biomass concentrations of microalgae cultures range from 0.5 to 18 g/L. As a result, a substantial quantity of water must be extracted to separate algal biomass (Kumar et al., 2019a). Two steps comprise the harvesting procedure: sedimentation/flocculation and dewatering. Microalgal cells have a very lower size range and, thus are impracticable to extract via conventional sedimentation. Centrifugation is the commonly used technology but it is a costly and energy-intensive process. Hence, there is a requirement for a secondary cost-effective process with the ability to harvest large-capacity culture. Flocculation is a process that can overcome such drawbacks and it involves the interaction between cell surface charges and flocculant charges. This process results in the generation of agglomerates in the medium which can be settled under the effect of gravity and can yield a concentrated algal slurry that contains at least 25% dry matter. For microalgae harvesting, numerous flocculation techniques have been documented; among these, chemical flocculation, bio-flocculation, and auto-flocculation have received the most research attention (Kumar et al., 2019a; Arora et al., 2021; Agarwalla and Mohanty, 2024). The economic and sustainable wastewater management along with proper recovery of nutrients from wastewater is a favorable outlook to mitigate the problem of water scarcity (Yadav et al., 2022).

Microalgae are increasingly recognized as a highly promising and sustainable long-term renewable resource. Algae with a high lipid content and rapid growth rate are chosen for a variety of applications across industries, including the production of biofuel, exopolysaccharides, biopolymers, and biofertilizers (Arora et al., 2021; Ramírez Mérida and Rodríguez Padrón, 2023). The increase in plastic usage in daily life leads to environmental pollution and these plastics are recalcitrant for degradation using microbes. Therefore, bioplastic can be an alternative to conventional plastics. These bioplastics raw materials can be obtained from biological sources like bacteria, microalgae, yeast, and transgenic plants. The biologically derived plastics are polyhydroxyalkanoates (PHA), polylactic acid, starch, and carbohydrates. These polymers can be extracted from biomass; extracellularly and intracellularly produced by microorganisms; and synthesized by bio-derivatives (Arora et al., 2021). Many microalgal strains are cultivated for polyhydroxyalkanoates production in different wastewater and stress conditions (Laycock et al., 2014; Wicker et al., 2022; Kusmayadi et al., 2023). Laycock et al. reported the production of 10 wt% of polyhydroxyalkanoates from *Spirulina platensis* in the presence of acetate and CO_2_ (Laycock et al., 2014). In another study, a photosynthetic consortium was cultivated in raw aquaculture effluent for polyhydroxybutyrate (PHB) production. The most significant amounts of PHB accumulation were seen under the high-intensity full-spectrum light treatment, which is correlated to biomass production, carbon utilization, and nutrient removal (Wicker et al., 2022). PHA also has applications in the medical sector such as scaffolds, tissue engineering, and surgical sutures. Additionally, microalgae can be used as biofertilizers when cultivated on wastewater (Arora et al., 2021). An experiment conducted by Das et al. (2019a) reported a yield of 650 mg L^–1^ of *Chlorella* sp. microalgae cultivated in municipal waste as a biofertilizer. Furthermore, the accumulated lipids in microalgae can be used as feedstock for biodiesel production (Hemalatha et al., 2019) while the whole biomass can be converted into bio-oil via thermochemical methods (Ravi Kiran et al., 2024). Also, the microalgae can undergo pretreatment for extraction of carbohydrates for bioethanol and biohydrogen production (Chokshi et al., 2016; Bhatia et al., 2021). In this line, the studies should be focussed on the integration of dairy wastewater bioremediation using microalgae, recycling of water, and biomass processing into value-added products or fuel.

## 2 Characteristics of dairy wastewater

### 2.1 Composition and properties of raw wastewater

Nowadays, the dairy industry wastewater is surveyed as one of the most polluted effluents in terms of BOD, COD, and total suspended solids (TSS). However, the volume of wastewater and pollution load is dependent on the type of products produced and the production process. The sterilized packaging unit of the Saras dairy factory processes a total of 1,00,000 L of milk/day, whereas the facility’s processing capacity is 5,00,000 L per day. Brar et al. reported that wastewater generated from dairy wastewater from the Saras dairy plant, Jaipur has a COD of 1,280 ± 226.47 mg L^−1^ and BOD of 245.95 ± 8.48 mg L^−1^. The total phosphate and nitrogen content of the dairy wastewater also has significant values of 19,583 ± 424 mg L^−1^ and 363.97 ± 23.93 mg L^−1^, respectively (Brar et al., 2019). Comparatively, the dairy wastewater at Jelgava, Latvia has a COD of 1,680 ± 20 mg L^−1^ and BOD of 1,196 ± 50 mg L^−1^, which is higher than the permissible limits. The wastewater contains nitrogen and phosphate of 115 ± 30 mg L^−1^ and 22 ± 05 mg L^−1^. Also, the wastewater reported the presence of lipids which was confirmed by Nuclear Magnetic Resonance (NMR) spectroscopy (Ekka et al., 2022). Qasim and Mane characterized the dairy wastewater of Pune City, Maharashtra, as having a COD of 8,960 ± 716.4 mg L^−1^ and BOD of 442 ± 3.1 mg L^−1^ (Qasim and Mane, 2013). A study reported that wastewater generated from yogurt and buttermilk dairy wastewater has less pollution load in terms of COD and BOD. A dairy factory in Erbil City generates 40–50 tons of yogurt and buttermilk every day. The COD value ranges from 0.986 to 1.132 g L^−1^ and BOD ranges from 0.6 to 0.8 g L^−1^ (Aziz and Ali, 2017). When the DW is released into the lakes and rivers without any treatment leads to eutrophication. This increases the growth of microorganisms that may deplete the dissolved oxygen in the water bodies. This makes the dairy sector one of the most notable contributors to the pollution of water bodies. pH is an important parameter in considering the quality of wastewater because microbial growth will depend on the pH of the wastewater. White wastewater produced after the cleaning of pasteurizers from both two Canadian dairy plants has an alkaline pH ranging from 8.23 to 12.45. However, the total solid from plant A (0.50 ± 0.04 g/L) was comparatively less than from plant B (3.12 ± 0.24 g/L), which signifies less dilution of later. Also, the alkaline and acidic wastewater were collected after second and fourth steps of the cleaning-in-place protocol and characterized for their chemical properties. The acidic wastewater generated from both plants A and B has very acidic pH (1.82 ± 0.06-plant A and 1.17 ± 0.01-plant B) with comparative electrical conductivity (5.35 ± 0.10 μS/cm -plant A and 14.25 ± 0.13 μS/cm -plant B). The comparative conductivity was observed due to significant calcium ions (177.04 ± 0.43 mg/L) reported from plant B acidic wastewater (Alalam et al., 2021). The pH of Yoruksut dairy wastewater has a slight acid-to-neutral range (6.75–7.71) while the total solid was less (1,200 mg/L) in March compared to May month (3,900 mg/L), exceeding the EPA limit (Aziz and Ali, 2017). Sawalha et al. characterized the dairy industry wastewater in Palestine and conducted an adsorption study using biowaste. Three samples were collected after pasteurization, cheese making, and washing process (soda washing and acid washing). The wastewater from different places was massively concentrated in terms of organics, chloride ions, pH, and TSS. However, the organics and TSS of cheese production wastewater were higher than those from the yogurt production process (Sawalha et al., 2022). TSS are crucial polluting indicator that is used for evaluating DW pollution and to measure the effectiveness of the wastewater treatment plant. The suspended matter in wastewater comes from viscous milk and small fragments of curd or flavorings (Garcha et al., 2016). The higher value of TSS and COD in cheese wastewater might be a result of whey protein, lactose, and fats (Sawalha et al., 2022). Whey wastewater has a high level of organic matter and nutrients, which can be utilized by microorganisms for their growth and metabolism. In another investigation, de Andrade et al. collected and analyzed the curd cheese whey for microalgal bioremediation. The whey has COD of 52,886 ± 269.25 mg L^−1^ with total nitrogen and phosphate of 1.56 ± 0.035 g L^−1^ and 0.66 ± 0.012 g L^−1^, respectively (de Andrade et al., 2023). In a study conducted by Bharadwaj et al., 52 microbes which include both bacteria and fungi have been identified and subjected to a screening process to determine their efficiency in degrading dairy wastewater. The genera *Serratia*, *Stenotrophomonas*, *Brachybacterium*, and *Cunninghamella* were reported for their activity in degrading dairy wastewater. The COD level of wastewater was reduced to 58%–72% using these three native genera (Bhardwaj et al., 2018). Overall, in both developed and developing nations, compliance with stringent environmental regulations has become obligatory for the discharge of effluents beyond the allowable limit. The initial physicochemical characteristics of different dairy wastewater collected is given in Table 1.

**TABLE 1:** Initial physico-chemical characteristics of different collected and synthetic dairy wastewater.

Collection location	Initial COD (mg/L)	Initial nitrogen (different forms) (mg/L)	Initial phosphate/phosphorous (different forms) (mg/L)	pH	Solids (mg/L)	TOC (mg/L)	References
Sarvottam Dairy Effluent (Gujarat, India)	2,593.33 ± 277.37	277.40 ± 10.75	5.96 ± 0.04	7.8	2,800 ± 20	116.23 ± 4.38	Chokshi et al. (2016)
Aochun Dairy Co., Ltd. (Foshan, Guangdong, China)	2,128 ± 12	121.0 ± 1.4	39.6 ± 3.2	9.31 ± 0.10	560 ± 6	—	Qin et al. (2016)
Local dairy industry (Saharanpur city, Uttar Pradesh)	2,843 ± 13	105 ± 3	36 ± 3	9.15 ± 0.2	1,586 ± 18	—	Chandra et al. (2021)
Wastewater from dairy products from Agro industries (Parana, Brazil)	190 ± 20	18.04 ± 0.50	2.63 ± 0.06	10.26 ± 0.25	60	7.42 ± 0.12	Melo et al. (2022)
DW (Amul dairy, Gujarat, India)	7,110	46.5	74.1	3.69	3,720	—	Kumar et al. (2019b)
DW, Pune City	8,960 ± 16.4	120.1 ± 2.5	—	7.10 ± 0.12	543.4 ± 5.2	—	Qasim and Mane (2013)
Synthetic Dairy wastewater	3,840	247.8	401.3	7	—	—	Singh et al. (2023)
Synthetic Dairy wastewater	3,600	160 ± 3 mg/L	180 ± 2.1	—	—	—	Mohanty and Mohanty (2023b)
Synthetic Dairy wastewater	3,600	158.69 ± 2.40	175.97 ± 1.81	7.0 ± 0.5	—	—	Kiran and Venkata Mohan (2022)
Synthetic Dairy wastewater	1,164	16.51	12.9	7	—	—	Divya Kuravi and Venkata Mohan (2021)
CPCB (1986)	250	50 (NH_4_-N) 10 (Nitrate)	—	5.5 to 9.0	100 (suspended)	—	Central Pollution Control Board (1986)

### 2.2 Evaluation of metals and organic matter present in the wastewater

The presence of organic matter like urea, carbohydrates, and fats also affects the quality of the wastewater. Various fatty acids were analyzed in wastewater from the dairy industry situated at Jelgava, Latvia. The wastewater comprises 65% hexadecanoic acid followed by 21% octadecanoic acid. Tetradecanoic acid was also present in wastewater in major amounts but oleic acid, linolenic acid, lauric acid, and linoleic acids were present in smaller concentrations. It was found that milk fatty acids majorly consist of saturated fatty acids. The presence of fatty acids in dairy wastewater offers a viable and cheap option for biodiesel production (Ekka et al., 2022). The quality and treatment efficiency of dairy wastewater also depend on the types of organic matter present in them because these compounds can attach to particulates and can cause abrasion, deposition, and clogging of membranes and filters during operations. The examination of trace organic chemicals found in the effluent of a dairy plant revealed the presence of common milk degradation products as well as compounds that may be linked to their synthetic or agricultural origins. The compounds that were found to be highest in the effluent are 1-Methyl-5-oxo-L-proline methyl ester (Verheyen et al., 2011). Zinc (Zn), cobalt (Co), copper (Cu), chromium (Cr), iron (Fe), and lead (Pb) are among the prevalent heavy metal pollutants detected in DW, and they are significantly considered as most critical global environmental problems (Table 2). Metals that are present in water bodies can persist for a prolonged amount of time or undergo biological transformations. Eventually, they accumulate throughout the food chain, presenting a significant threat to the ecology if not adequately removed. Removing heavy metals from wastewater is challenging due to their resistance to chemical or biological treatment. The chloride, iron, and fluoride concentrations of 199, 5.17, and 4.833 mg L^−1^ were addressed by Kumar et al. in raw dairy wastewater collected from Amul Dairy, Gujarat, India (Kumar et al., 2019b). Also, the dairy eluent collected from Pune City has a chloride level of 186.4 ± 3.4 mg L^−1^ (Qasim and Mane, 2013), which is lower than EPA regulations. The DW obtained from Sarvottam Dairy effluent contained a high amount of sodium (345.65 mg/L). While little amount of nickel, copper, cobalt, iron, and chromium was observed in DW (Chokshi et al., 2016). The elevated levels of sodium and chloride are attributed to the extensive use of alkaline cleaning agents in dairy facilities. Aluminum can come from aluminum sulfate which is frequently employed in water treatment facilities for the purpose of clarifying the water (Qasim and Mane, 2013). Trace elements such as copper and zinc, as well as other heavy metals including cadmium, arsenic, chromium, and mercury, may be found in dairy wastewater. These elements enter the wastewater via therapeutic substances and organic molecules from pesticides (Qasim and Mane, 2013).

**TABLE 2:** Comparison of metals present in the different dairy industry generated wastewater. Every metals is reported in mg/L.

Wastewater collection points	K	Na	Cl	Cr	Zn	Ca	Cd	Mg	Al	Mn	Ni	Cu	Co	Fe	Pb	References
Mean of 15 plants	46.6	544	—	—	—	48.9	—	20.9	139	163	36	8	2	725	—	Danalewich et al. (1998)
Yoruksut dairy factory wastewater (January 2016)	—	—	53.98	—	—	—	—	—	—	12.5	—	—	—	—	—	Aziz and Ali (2017)
Yoruksut dairy factory wastewater (March 2016)	—	—	70	—	—	—	—	—	—	10.2	—	—	—	—	—	Aziz and Ali (2017)
Yoruksut dairy factory wastewater (May 2016)	—	—	94.97	—	—	—	—	—	—	26.6	—	—	—	—	—	Aziz and Ali (2017)
Dairy products	4.39 ± 1.91	0.067 ± 9e^−5^	—	—	—	29.86 ± 0.13	—	2.72 ± 0.01	0.022 ± 0.00082	—	—	—	—	—	—	Melo et al. (2022)
Sarvottam DW	0.30	345.65	—	0.50	0.30	38.10	—	28.45	0.30	1.25	0.80	0.30	0.65	0.30	0.65	Chokshi et al. (2016)
DW (Amul)	—	—	—	—	—	—	—	—	—	—	—	—	—	5.17	—	Kumar et al. (2019b)
Acid cleaning wastewater Plant A	0.76 ± 0.09	155 ± 13	—	—	—	12.22 ± 1.45	—	1.89 ± 0.17	—	—	—	—	—	—	—	Alalam et al. (2021)
Acid cleaning wastewater Plant B	1.57 ± 0.06	23 ± 4	—	—	—	177.04 ± 0.43	—	7.07 ± 0.05	—	—	—	—	—	—	—	Alalam et al. (2021)
Alkaline cleaning wastewater Plant A	9.41 ± 0.26	10,665 ± 191	—	—	—	45.15 ± 1.76	—	0.82 ± 0.10	—	—	—	—	—	—	—	Alalam et al. (2021)
Alkaline cleaning wastewater Plant B	6.48 ± 0.07	4,033 ± 46	—	—	—	18.81 ± 1.98	—	0.23 ± 0.03	—	—	—	—	—	—	—	Alalam et al. (2021)
DW	5.26	—	186.4 ± 3.4	0.061	—	—	0.065	—	—	0.32	—	0.061	—	0.065	0.040	Qasim and Mane (2013)
CPCB (1986)	—	—	—	—	5.0	—	2.0	—	—	2	3.0	3.0	—	3	0.1	Central Pollution Control Board (1986)

## 3 Dairy wastewater treatment technologies

**FIGURE 1:**
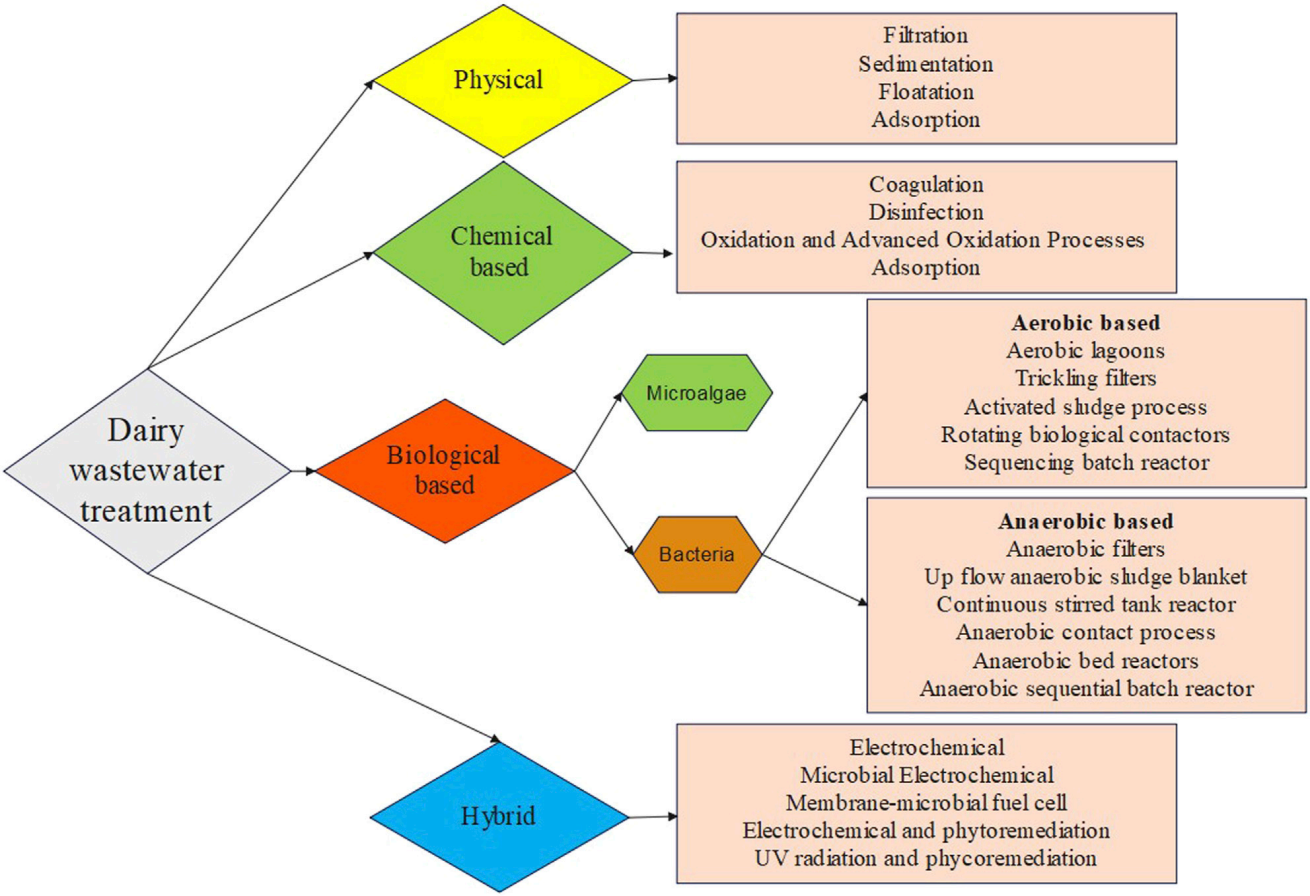
Various dairy wastewater treatment technologies.

Dairy wastewater has the potential to serve as a nutrient source for the production of biomass and recovery of value-added products. The complex characteristics of dairy wastewater make it a challenging category of industrial wastewater, namely, because of the high-fat content and high levels of COD. Dairy effluent is treated using four major different approaches: coagulation, membrane technology, biological methods, and hybrid methods in previous studies (Table 3; Figure 1). Every technology has its advantages and disadvantages in treating dairy wastewater (Table 4). The selection of technology and reactor required for the design of an effluent treatment plant is determined by the availability of land, infrastructure, and the efficiency of focused treatment (Krishna B et al., 2022).

**TABLE 3:** Treatment technologies reported for Dairy Wastewater.

Technology	Findings	References
*Physical*		
Reverse osmosis and Nanofiltration	The RO and NF membranes demonstrated exceptional performance by removing 99.7% and 98% of the COD, respectively, from a feed COD concentration of 40 and 450 mg/L for NF feedwater and 5,000 and 10,000 mg/L for RO feedwater.	Turan (2004)
Microfiltration + nanofiltration Microfiltration + reverse osmosis	MF + RO system was more effective in retaining organic matter and total solids.	Bortoluzzi et al. (2017)
Nanofiltration	A high and stable flux was observed in DW treatment by NF under an extreme enhanced shear rate.	Luo et al. (2012)
Ultrafiltration	The results indicated that the flux of UF was greater at pH 4.6 than at pH 8, as the resistance of the fouling membrane was lower at the isoelectric point of protein (pH 4.6) during UF operation.	Gong et al. (2012)
*Chemical*		
Electro-chemical (EC) method using iron electrode	The efficiencies of COD, TS, TN, and turbidity removal were determined to be 70%, 48.2%, 92.75%, and 99.8%, respectively, under optimal conditions.	Kushwaha et al. (2010)
Electrocoagulation using mild steel electrodes	The findings showed that the elimination efficiency of the chemical oxygen demand (COD) and oil grease was 98% and 99%, respectively.	Şengil and özacar (2006)
Electro-Fenton process using iron electrode	The optimal conditions for achieving a 93.93% COD removal were a reaction time of 90 min, a current density of 56 mA/cm^2^, pH 7.52, an H_2_O_2_/DW volumetric ratio of 0.898 mL/L, and an H_2_O_2_/Fe^2+^ molar ratio of 3.965. Similarly, the optimal conditions for achieving a 97.32% removal of colour were a reaction time of 86 min, a current density of 55.1 mA/cm^2^, pH 7.48, an H_2_O_2_/DW volumetric ratio of 0.907 mL/L, and an H_2_O_2_/Fe^2+^ molar ratio of 3.987.	Davarnejad and Nikseresht (2016)
Electrocoagulation using aluminium electrodes	The chemical oxygen demand (COD) was reduced by up to 61% while the removal of phosphorus, nitrogen contents, and turbidity were 89, 81% and 100%, respectively.	Tchamango et al. (2010)
Coagulation with three chemicals- aluminium sulfate, calcium hydroxide, and iron chloride	The calcium hydroxide at the low dose range provides the efficient removal of suspended matter (94%) and total phosphorus (89%) accompanied by an average elimination of chemical oxygen demand (COD), total Kjeldahl nitrogen (TKN-N), and microbial community and generates less sludge compared to aluminium sulfate and iron chloride.	Hamdani et al. (2005)
Coagulation with inorganic (Alum and ferrous sulphate) and polymeric coagulants (polyacrylamide [PAA] and polyferric sulphate [PFS])	The data indicate that alum had more efficacy in removing turbidity and COD, with a removal rate of 95% for turbidity and 68% for COD, compared to ferrous sulphate which achieved a removal rate of 95% for turbidity and 62% for COD. When Alum, in combination with PFS and PAA as coagulant aids, was used, a significant reduction of 82% in COD was achieved with a modest dosage of alum at 100 mg/L.	Loloei et al. (2014)
Coagulation using poly aluminum chloride	The chemical coagulation technique obtained the highest removal efficiency of pollutants (BOD_5_) and chemical oxygen demand (COD) at an initial pH of 8 and a coagulant dosage of 100 mg/L in 60 min.	Bazrafshan et al. (2016)
Ultraviolet radiation	A UV fluence of 1892.7 mW s·cm^−2^ reduced bacteria colony-forming units in an anaerobic digester and polyhydroxyalkanoate reactor of dairy wastewater by 99% and 100%, respectively with reduced dissolved organic matter.	Passero et al. (2014)
Combined aerated electrocoagulation	Electrocoagulation was determined to be effective at neutral pH conditions, and its efficacy was seen to rise when the applied voltage was raised. The highest COD removal effectiveness of 86.4% was achieved while using the Al-Fe electrode combination with aeration for a reaction duration of 120 min, an initial pH of 7, and a voltage of 5 V.	Akansha et al. (2020)
Biological (Aerobic and anaerobic)		
Activated sludge	When at least 45.4 kg O_2_ d^-1^ (30/45) were provided, COD removal efficiencies were always in the range of 88%–94% but decreased to about 70% under aeration regimes 15/45 and 30/60.	Tocchi et al. (2012)
Membrane sequencing batch reactor system	BOD removal of 97%–98% and nitrogen removal of 96% were observed.	Bae et al. (2003)
Membrane bioreactor technology	Aerobic MBR may reduce the BOD_5_ level in DWW by up to 99% and the ammonium levels by up to 99.9%.	Stepanov et al. (2019)
Aerated filtration	COD and BOD were reduced to appx. 95%. Ammonical nitrogen was reduced from 9 to 10.2 mg L^1^ to <0.3 mg L^1^ and total phosphorus from 20 to 21.2 mg L^1^ to <4 mg L^1^.	Scott and Smith (1997)
Upflow anaerobic filter	COD removal efficiency of about 80% was observed at a maximum organic loading rate of 17 g COD L ^1^ d^1^.	Rajagopal et al. (2013)
Anaerobic filter reactor	The organic loading rates were between 5 and 6 kg COD/m^3^ d, with COD removal being higher than 90%.	Omil et al. (2003)
Downflow-upflow hybrid reactor	The process successfully converted 98% of the chemical oxygen demand into biogas, while also removing over 90% of ammonia and total phosphorus.	Malaspina et al. (1996)
Up-flow anaerobic sludge blanket reactors	COD reduction of 96.3% in 3 h was noticed in up-flow anaerobic sludge blanket reactors.	Passeggi et al. (2012)
Upflow anaerobic sludge blanket reactor	COD and BOD removal of 77% and 87%, respectively was achieved in the reactor for the treatment of dairy wastewater.	Kavitha et al. (2013)
Anaerobic treatment	At an organic loading of 1 g/L and a retention duration of 72 h, the COD reduction % at 35°C without additional seeds was reported to be 50%. While the COD removal efficiency of 83.33% with the addition of seeds was observed.	Dawood et al. (2011)
Microalgal based treatment		
*Acutodesmus dimorphus*	The COD of dairy effluent decreased by more than 90% (2,593.33 ± 277.37 to 215 ± 7.07 mg/L) using *Acutodesmus dimorphus* following cultivation for 4 days.	Chokshi et al. (2016)
*Monoraphidium* sp. KMC4	The highest COD removal efficiency of 93.4% was achieved in 12.5% of simulated synthetic dairy wastewater.	Singh et al. (2023)
*Monoraphidium* sp. SVMIICT6	COD, nitrates, and phosphates removal efficiencies of 75%, 85%, and 60%, respectively were observed.	Divya Kuravi and Venkata Mohan (2022)
Mixed microalgae	COD reduced from 1746 mg L^−1^ to 174 mg L^−1^ by the end of the 6th day with the treatment efficiency of 90%.	Hemalatha et al. (2019)
*C. pyrenoidosa*	About 80%–85% of phosphorus and 60%–80% of nitrogen were removed from the dairy wastewater.	Kothari et al. (2012)
Hybrid		
Coagulation and adsorption	The removal efficiency of most pollutants from raw dairy wastewater was high, still the coagulation process alone was not able to meet the discharge standards. The combination of adsorption in the treatment process enhanced the pollutant removal efficiency.	Bazrafshan et al. (2016)
Coagulation and electro-Fenton	The removal efficiency of 90.3%, 87.25%, and 87% for COD, BOD_5_, and total suspended solids, respectively was observed.	Zakeri et al. (2021)
Catalyst-less and mediator-less membrane microbial fuel cell	The findings demonstrate that removal efficiency rises in concordance with operating duration, rising from 78.21% to 90.46% for COD and from 61.43% to 81.72% for BOD_5_.	Mansoorian et al. (2016)
Anaerobic process (up-flow anaerobic sludge blanket reactor) and advanced oxidation processes (AOPs)	The anaerobic reactor had a maximum loading rate of 19.2 kg COD/m^3^ day and a COD removal rate of 84% at this OLR. Anaerobically treated wastewater at 19.2 kg COD/m^3^ day underwent secondary solar photocatalytic oxidation. The optimal pH and catalyst loading for solar photochemical oxidation are 5 and 300 mg/L, respectively. TiO_2_-based secondary solar photocatalytic oxidation eliminated 62% of COD from initial anaerobic treatment. Anaerobic and solar photocatalytic treatment together removed 95% of dairy wastewater COD.	Rajesh Banu et al. (2008)
Aerated electrocoagulation and phytoremediation	97.9% COD reduction was observed in dairy wastewater with combined technology.	Akansha et al. (2020)
UV Irradiation and sodium hypochlorite and microalgae	Pretreatment of dairy wastewater by UV and NaClO was found to be feasible for large-scale cultivation. The highest biomass productivity and lipid productivity of *C. vulgaris* could reach 0.450 g L^−1^ day^−1^ and 51 mg L^−1^ day^−1^.	Qin et al. (2014)

**TABLE 4:** Comparative table on advantages and disadvantages of various treatment technologies.

Technology	Merits	Demerits
Chemical Technology	• Natural coagulants are safe to use	• May require pH control
Physical technology	• Easy integration with biological system • Low energy consumption and cost effective treatment	• Energy-intensive • Prone to membrane fouling Produces high concentrated effluent • limited contaminant removal like suspended particles and space requirement for sedimentation and aeration
Biological Technology (Aerobic)	• Low energy usage • Compact design (Aerobic and Membrane filtration) • Effective at removing complex compounds (activated sludge) • Low resistance to environmental shock (activated sludge)	• Accumulation of sludge (activated sludge)
Biological Technology (Anaerobic)	• Suitable for high COD stream • Higher resistance to use environmental shock due to granular sludge	• Ineffective at treating high FOG content • Prone to accumulation of fats on the sludge blanket
Biological Technology (Microalgae)	• Nutrient recovery in the form of valuable biomass • Carbon dioxide sequestration	• Probable contamination in large scale

### 3.1 Physical treatment technology

As dairy industrial wastewater contains high amounts of dissolved organic matter, membrane technology offers many advantages, including a high degree of removing the dissolved, colloidal, and particulate matter; selectivity in the removal of contaminants based on size; and the possibility for extremely compact treatment facilities (Turan, 2004). The dairy industrial wastewater is initially screened to remove the large debris that can clog the further treatment pipelines. The delay in the screening step may increase the COD of wastewater due to solid solubilization (Zainab et al., 2019). Many studies have been reported earlier for the treatment of dairy wastewater using reverse osmosis (RO) (Turan, 2004), nanofiltration (NF) (Turan, 2004; Luo et al., 2012), and ultrafiltration (UF) (Gong et al., 2012; Tayawi et al., 2023). RO membrane was used for the treatment of high-strength dairy industry wastewater (5,000 and 10,000 mg/L-COD) and showed excellent performance by removing 99.7% COD. While the nanofiltration membrane was used for low-strength wastewater (40 and 450 mg/L-COD), and showed a COD removal efficiency of 98%. The fouling of nanofiltration and reverse osmosis were also investigated by Turan (2004). The reduction in filtration efficiency resulted from an increase in the fouling layer and concentration polarization layer. The fouling behavior of dairy wastewater treatment by nanofiltration was investigated in another study by Luo et al. A rotating disk laboratory module with high shear and pressure was applied to treat the dairy wastewater using the NF270 membrane. The flux profile and permeate quality were not significantly affected by the presence of lipids, although adsorption fouling was marginally increased. Concentration polarisation was reduced by increased shear rates, which resulted in higher permeate fluxes and reduced permeability loss. The inorganic ions form aggregates with milk proteins, causing negligible inorganic fouling and alkaline cleaning could remove surface fouling. A high and stable flux was observed in DW treatment by NF under an extremely enhanced shear rate (Luo et al., 2012). Also, integrated membrane systems process in sequential form showed better performance in terms of COD, TOC, and nutrient removal. The integrated systems consist of the sequential use of microfiltration (MF) and nanofiltration (NF) and MF and reverse osmosis (RO) under varying pressures to treat dairy effluent. The MF + NF system resulted in a 100% reduction in turbidity, 96% in colour, 58% in total Kjeldahl nitrogen (TKN), and 51% in COD. The MF + RO system resulted in a 100%, 100%, 94%, and 84% reduction in turbidity, colour, total dissolved nitrogen, and TOC, respectively. Consequently, the MF + RO system was more effective in retaining organic matter and total solids (Bortoluzzi et al., 2017). Floatation is also a technique used for the treatment of dairy wastewater but merged with chemical-based coagulants for better efficiency (Pereira et al., 2020). Adsorption is a method of wastewater treatment that effectively removes a significant quantity of non-degradable organic components from wastewater. The most often utilized adsorbent is activated carbon. Low-cost adsorbents such as rice husk, coal fly ash, and straw dust are used for wastewater treatment (Sinha et al., 2019). In a previous study, activated charcoal achieved a maximum removal efficiency of 65% for COD and 67% for BOD in dairy effluent (Kanawade and Bhusal, 2015). With the advantages of low energy consumption and cost-effective treatment, the physical treatment technology set the major disadvantages of limited contaminant removal like suspended particles and space requirement for sedimentation and aeration (Yonar et al., 2018).

### 3.2 Chemical treatment technology

Chemical treatment includes processes such as pH balance and reagent oxidation, which are beneficial for the removal of soluble contaminants and colloids in wastewater. The dairy industry effluent exhibits a pH range of 4.7–11 and extreme values can have adverse effects on microbiological cells in biological reactions and increase the corrosion of pipelines. Consequently, it should be adjusted to mitigate its harmful effects (Zainab et al., 2019). One approach to treat DW is electrochemical treatment, which entails the utilization of an iron electrode to treat simulated dairy effluent. It is highly effective in the treatment of nutrient-rich wastewater by reducing the COD and oil–grease in the aqueous phase (Şengil and özacar, 2006). In another study by Kushwaha et al. (2010), COD removal efficiency of appx. 70% was observed using the electrochemical treatment method with iron electrodes. A combination of electro-coagulation, electro-floatation, and electro-oxidation mechanisms were hypothesized as the main routes for COD depletion in dairy effluent. In another electrochemical-based treatment, H_2_O_2_/Fe^2+^ molar ratio and H_2_O_2_/dairy wastewater ratio (DW) (mL/L) obtained the maximum COD removal of 93.93% within 90 min (Davarnejad and Nikseresht, 2016). On the other hand, aluminum electrodes in the electro-coagulation process removed 60% COD in dairy wastewater (Tchamango et al., 2010) while only 39% COD was removed by chemical coagulation (Hamdani et al., 2005). Dairy wastewater also contains major amounts of fat, oil, and grease which are generated during unskinned milk production. The separation of fats from the wastewater can be done by increasing the temperature. Similarly, the proteins and lipids components in wastewater can be eliminated by the coagulation process. The flotation process by dissolved air technique is more effective due to the reduction of organic load, protein, and lipid colloids through the use of flocculants and coagulants. This method utilizes synthetic and costly compounds, which result in environmental issues. The use of organic coagulants like polyacrylamide [PAA] and polyferric sulphate [PFS] reduced the COD and turbidity level of dairy wastewater, with less or no environmental damage (Loloei et al., 2014). The maximum BOD_5_ and chemical oxygen demand removal efficiency was achieved at pH 8 and with poly aluminum chloride in 60 min in dairy wastewater (Bazrafshan et al., 2016). Also, the utilization of UV irradiation helps in reducing the microbial load and dissolved organic method of dairy wastewater (Passero et al., 2014). In a study conducted by Qin et al., UV irradiation and sodium hypochlorite both were tested for their efficiency in the treatment of dairy wastewater. The COD, total phosphorous, and total Khejdhal nitrogen displayed a slight reduction in their level after UV treatment but increasing values were observed in ammonium nitrogen. A similar trend was observed in the case of sodium hypochlorite treatment (Qin et al., 2014). In another study, a combined aerated electrocoagulation process also showed a COD removal efficiency of 86.40% in the case of Al-Fe electrode combination with aeration at optimized conditions (Akansha et al., 2020). The chemical-based treatment has the advantage of removing a wide range of contaminants, including dissolved and colloidal substances within less time duration. Despite this advantage, this treatment process has major setbacks in sludge production and disposal, handling and storage of potentially hazardous chemicals, and the treatment cost due to expensive chemicals (Mohammed Bello et al., 2019).

### 3.3 Biological treatment technology

The biological treatment process includes the use of microorganisms to reduce the organic load present in the wastewater. The physical condition of the treatment system will depend on many parameters like pH, temperature, and oxygen amount, which need to be controlled to avoid the death of the microbial community for treatment. Certain nutrient loads should not exceed the tolerance level of microbes before the treatment. Also, there can be a presence of heavy metals in the dairy wastewater, which can damage the cells during the treatment, and reduce the efficiency (Ramsuroop et al., 2024). Aerobic and anaerobic are two types of biological treatment methods (Goli et al., 2019). Many studies have employed individual aerobic and anaerobic treatment technology and numerous investigations have used combined strategies to overcome the limitations of individual processes (Goli et al., 2019; Ramsuroop et al., 2024).

The aerobic technique reduces the biological oxygen demand as well as phosphorous and nitrogen content in dairy wastewater. This process is also effective in removing the fats from the wastewater. The odor of wastewater is reduced when the ammonium nitrogen is converted to nitrates. In addition, the aerobic procedure will require aeration which requires high energy demand. Activated sludge treatment is one of the aerobic treatment methods, which employs the introduction of microbes in the wastewater. The microbes are then isolated using a clarifier or filter, while a fraction of the sludge is returned to the reactor (Goli et al., 2019). Research has shown that activated sludge (including both bacterial and protozoan) was reported to be successful in decreasing organic compounds in dairy wastewater, the best performance was obtained at 45.4 kg O_2_ d^−1^ (Tocchi et al., 2012). This process had the advantages of easy operation and a light footprint (Goli et al., 2019). Low environmental shock tolerance and toxin buildup are common issues in activated sludge operations. Additionally, sludge settling might hinder biomass recovery. Granular sludge, which generates solid spherical granules from microbes and flocs, has been used to address these shortcomings. These granules have enhanced shock resistance and settling qualities. Another drawback of activated sludge systems is the disposal of sludge (Ramsuroop et al., 2024). Sequencing batch reactors (SBRs) combine many processes in one bioreactor (Goli et al., 2019). These phases are sequential: filling, reacting, settling, decanting, and idling. Filling involves adding microbe-containing DW and microbes. The reaction step may include aerobic and anaerobic cycles. To do this, aeration and no aeration can be performed. At the settling stage, aeration and mixing are halted to allow suspended particles to separate from the treated water. In the decanting step, the supernatant fluid (treated wastewater) is removed. The idle stage is particularly important in multi-reactor systems with a delay between filling stages (Goli et al., 2019; Ramsuroop et al., 2024). Studies have shown that SBRs may decrease COD levels by as much as 90%, whereas COD concentration varies from 400 to 2,500 mg/L. One investigation has successfully treated dairy wastewater using a hybrid up-flow–downflow reactor, maintaining stability even with an average organic loading rate of 10,000 mg COD/L/day. This system showcases the versatility of an SBR system by including both downflow pre-acidification chambers and up-flow methanation chambers. The process successfully converted 98% of the chemical oxygen demand into biogas, while also removing over 90% of ammonia and total phosphorus (Malaspina et al., 1996). Additionally, research has been conducted using a sequencing batch reactor (SBR) in conjunction with membrane filtration to address these constraints. Nevertheless, the use of a membrane presents the added obstacle of membrane fouling, necessitating the implementation of further measures to minimize this potential problem. One disadvantage of SBRs is that they operate as a batch system, meaning that the reactors need to be loaded, unloaded, and cleaned for each batch. This leads to decreased production compared to a continuous system (Ramsuroop et al., 2024). In a membrane bioreactor, the membrane is submerged within the reactor and another configuration is one where the membrane is placed on the exterior of the reactor with a recycle loop. It has been reported that an aerobic MBR may reduce the BOD_5_ level in DWW by up to 99% and the ammonium levels by up to 99.9% (Stepanov et al., 2019). In another investigation, aerobic MBR treated ice-cream wastewater with high levels of contaminants, namely, 13,300 mg COD/L and 6,500 mg BOD_5_/L. This treatment resulted in a reduction of over 95% in COD and BOD_5_ levels, an 80% decrease in TP, and a decrease of over 96% in TN (Scott and Smith, 1997). Like other membrane filtering systems, the primary concerns for a practical MBR system are the cost of the process, membrane fouling, and methods to manage fouling (Goli et al., 2019). Other types of aerobic treatment reactors include tricking filters (Goli et al., 2019) and rotating biological contractors (Ramsuroop et al., 2024). The average treatment efficiency of trickling filters was 87.3%, 78.3%, and 27.9% without recirculation for COD, BOD, and total phosphorous while this treatment efficiency increased when recirculation was applied (Zyłka et al., 2018). A significant concern is that trickling filters may get obstructed by the accumulation of ferric hydroxide and carbonates, resulting in a decrease in the activity of microbes. When there is an excessive amount of dairy wastewater, the fluid will get obstructed by dense biological and fat films (Goli et al., 2019). The design of the rotating biological contactor (RBC) involves the use of circular discs, which promote the growth of a biofilm on its surface to allow the oxidation of organic molecules in the wastewater (Kadu et al., 2013). The maximum COD removal efficiency of 94% in dairy wastewater was observed using a 3-tank biological contactor reactor (Asha and Elakkiya, 2014). The RBC method has some advantages over the ASP when it comes to the treatment of dairy wastewater. The primary advantages of the RBC process are little power consumption, straightforward operation, and minimal maintenance requirements. However, in comparison to the trickling filter, RBC requires less area for treatment and incurs reduced running expenses (Goli et al., 2019).

While the anaerobic treatment is mainly focused on decreasing the amount of organic matter and treating high concentrations of organic substances in wastewater. Anaerobic treatment can generate methane from wastewater that is rich in organic matter. Anaerobic technology is often used in anaerobic filters, sludge blanket reactors, and packed bed digestors (Goli et al., 2019). In a study conducted by Rajagopal et al. (2013), a reduction in 80% COD was achieved when treating a DW stream using anaerobic filters. Contrarily, this technique does not show effective results in removing the fat, oil, and greases (FOG) from high-fat-containing dairy wastewater (Omil et al., 2003). This highlights the requirement of alternative technology for treating high organic-containing wastewater. Anaerobic packed bed reactors are another technology that was highly effective at reducing COD, BOD, and suspended solids up to 96%, 93%, and 90%, respectively. Up-flow anaerobic sludge blanket reactors (UASBRs) are one of the most used for the treatment of dairy wastewater. UASB reactors are used for treating wastewater that has COD up to 40 g/L. The reactor showed a COD reduction of 96.3% in 3 h (Passeggi et al., 2012). A major hurdle involved in these reactors is the accumulation of organic matter in the sludge blanket, making the difficult for anaerobic bacteria to break down the FOG. Implementing a hydrolysis stage before the reaction or increasing the reaction time of the reactor would resolve the difficulty (Ramsuroop et al., 2024). Another important treatment method is anaerobic digestion, 95% of the organic load in a waste stream can be turned into biogas (methane and carbon dioxide), while the rest is used for cell growth and maintenance. In addition, a small amount of sludge is generated in the anaerobic digestion process, reducing the difficulties related to sludge removal. Anaerobic digestion (AD) systems need nutrients, such as nitrogen and phosphorus, at levels much lower than those required by aerobic systems. One of the simple designs for AD design is a stirred tank reactor. Continuous stirred-tank reactors are often used for treating highly concentrated effluents, especially those containing a significant amount of suspended solids and chemical oxygen demand values over 30,000 mg/L. Biomass is not retained in this reactor, which means that the hydraulic retention time (HRT) and sludge retention time (SRT) cannot be distinguished. Consequently, extensive retention durations are required, based on the growth rate of the slowest-growing bacteria involved in the digesting process (Goli et al., 2019). The up-flow anaerobic sludge blanket reactor is a very popular technique used for the treatment of wastewater. The advantage involved in an up-flow anaerobic sludge blanket reactor (ASBR) is less sludge production compared to an aerobic treatment system due to the slow growth rate of anaerobic organisms and good removal efficiency is achieved even at high loading rates and low temperatures. The procedure involves the use of anaerobic microorganisms in a single tank to treat wastewater, resulting in the near-total elimination of organic contaminants, solids, and oil and grease (Sinha et al., 2019). COD and BOD removal of 77% and 87%, respectively was achieved in the reactor for the treatment of dairy wastewater. This technology removed the suspended solid and chlorides efficiently at the end of the treatment period (Kavitha et al., 2013). The ASBR is a recently created batch reactor system that integrates the processes of digestion and the separation of particulates into a single vessel. The treatment of wastewater by anaerobic sequencing batch reactors involves four sequential steps: feeding, reaction, settling, and removal of treated wastewater. This form of reactor is widely used because of its notable advantages, such as its simplicity, effective quality control of wastewater, less settling time, and versatility in treating various types of effluents. Nevertheless, a significant drawback of ASBR is its suboptimal performance under heavy load conditions (Sinha et al., 2019). At an organic loading of 1 g/L and a retention duration of 72 h, the COD reduction % at 35°C without additional seeds (pre-prepared culture media from synthetic milk waste and sewage) was reported to be 50%. While the COD removal efficiency of 83.33% with the addition of seeds was observed (Dawood et al., 2011). Also, aerobic–anaerobic combined process allows the complete remediation of dairy wastewater as every stage focuses on different contaminants in the wastewater. The aerobic process reduces the ammonium, phosphate, hydrogen sulfide, and BOD of the wastewater while the anaerobic process reduces the COD and nitrate concentration in the effluent (Ramsuroop et al., 2024).

### 3.4 Hybrid technology for dairy wastewater treatment

Biological approaches are often regarded as the most efficient means of treating dairy wastewater. Among these methods, aerobic systems are simpler to manage and regulate, while anaerobic systems generate less sludge and use less energy. It is advisable to construct a combined process that is particularly designed to meet the minimal criterion for discharging effluent (Sinha et al., 2019). Many studies have used the hybrid or combined process for the effective treatment of dairy wastewater. Bazrafshan et al. used an inorganic prepolymerized-based coagulation and adsorption process on modified dried activated sludge for dairy wastewater treatment. The removal efficiency of most pollutants from raw dairy wastewater was high, still the coagulation process alone was not able to meet the discharge standards. The combination of adsorption in the treatment process enhanced the pollutant removal efficiency (Bazrafshan et al., 2016). Another superior combination of chemical coagulation with the electro-fenton process was used by Zakeri et al. for the treatment of dairy wastewater. The removal efficiency of 90.3%, 87.25%, and 87% for COD, BOD_5_, and total suspended solids, respectively was noticed (Zakeri et al., 2021). The catalyst-less and mediator-less membrane microbial fuel cell is a novel approach that allows for the simultaneous treatment of dairy sector effluent and the production of bioelectricity. In a study conducted by Mansoorian et al., two chambers, namely, an anaerobic anode and an aerobic cathode compartment were divided by a proton exchange membrane for dairy wastewater treatment. The findings indicate that the removal efficiency for COD improves from 78.21% to 90.46% and for BOD_5_ it increases from 61.43% to 81.72% with increasing time (Mansoorian et al., 2016). The ultraviolet (UV) photocatalytic treatment has the benefit of further eliminating organic compounds in wastewater, while its effectiveness is limited to low-strength effluent. Utilizing solar radiation for wastewater treatment shows potential for areas with abundant light. The treatment of wastewater with a combined anaerobic process (up-flow anaerobic sludge blanket reactor) and advanced oxidation processes (AOPs) hold a promising route toward efficient wastewater treatment. The combination of anaerobic and solar photocatalytic treatment achieved a 95% reduction in COD levels in the dairy effluent (Rajesh Banu et al., 2008). Electrocoagulation is a popular technique for treating water and wastewater due to its combination of coagulation, flotation, and electrochemistry. The addition of air during the electrocoagulation process has an enhanced effect on reducing the COD of wastewater. Studies also showed an effective result when aerated electrocoagulation is combined with phytoremediation, 97.9% COD reduction was observed in dairy wastewater (Akansha et al., 2020). Another study combines the use of UV irradiation, and sodium hypochlorite (NaClO) as a pretreatment step before microalgae-based treatment of dairy wastewater. The highest biomass productivity and lipid productivity of *C. vulgaris* reached 0.450 g L^−1^ day^−1^ and 51 mg L^−1^ day^−1^ in dairy wastewater, respectively (Qin et al., 2014). From this, it can be concluded that combined hybrid technology with microalgae produced significant biomass with high-value product accumulation for various applications.

## 4 Role of microalgae in dairy wastewater treatment

### 4.1 An overview of the literature

**FIGURE 2:**
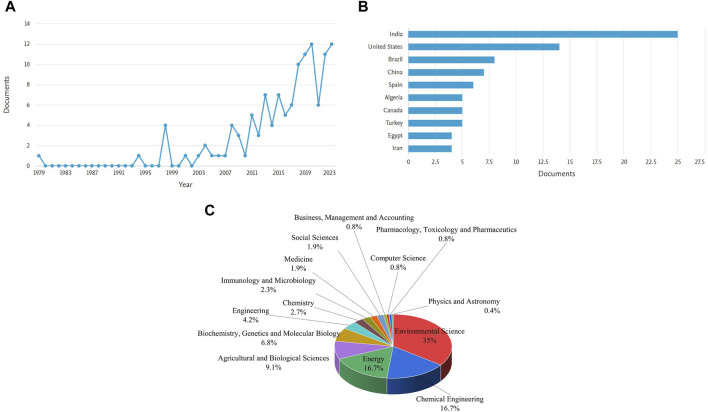
Scientometric analysis obtained from scopus database for dairy wastewater treatment **(A)** Distribution of articles **(B)** countries active in the research area **(C)** research field area.

It was observed that research on dairy wastewater treatment was started initially in 1996 and started to increase tremendously from the 20th century. The publications of 12–24 documents on the treatment of dairy wastewater were published from 2019 to 2023 while only 3–7 documents were submitted from 2014 to 2018. A total of 127 research articles have been published as of the retrieval date of the data. India emerged as the market leader in the dairy industrial sector and its treatment, as evidenced by its increased involvement in this research domain. Guangzhou Institute of Energy Conversion has performed major work in this domain area of dairy wastewater treatment. With the increase in industrialization and urbanization, DW was listed among the polluted effluents. Dairy wastewater, on the other hand, is one of the most extensively researched and acceptable nutrient mediums that is utilized in the production and growth of microorganisms because it contains adequate amounts of phosphate and nitrogen sources (Chokshi et al., 2016). There is a notable amount of studies being conducted at present to integrate microalgae with wastewater treatment. This shows that study in this area is becoming progressively more prevalent. It was also noted that over 34.90% of the study’s research was conducted in the field of Environmental science. This was followed by the fields of Chemical Engineering (16.73%), Energy (16.73%), Agricultural and Biological Sciences (9.12%), Biochemistry, Genetics and Molecular Biology (6.84%). This highlights that the field of dairy wastewater treatment primarily centers around the areas of Environmental Science, Chemical Engineering, and Energy (see Supplementary Tables S1–S4; Figure 2).

### 4.2 Keyword co-occurrence analysis

**FIGURE 3:**
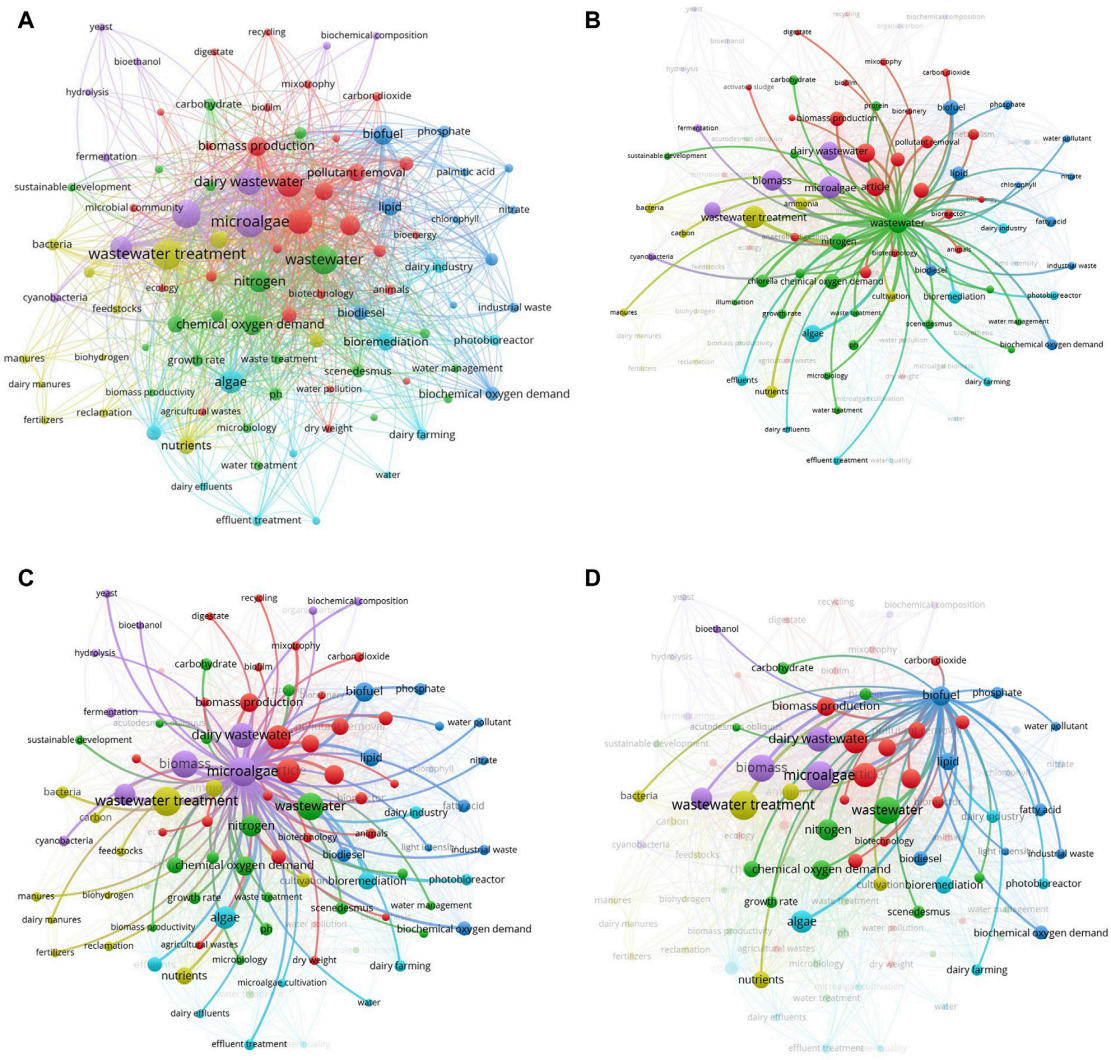
Significant keywords were observed in dairy wastewater treatment.

The search terms used throughout this area provide an in-depth understanding of disciplines that primarily focus on a certain domain. A network of interconnected keywords performs as an actual illustration of the interaction between them. The current study included the quantitative methods of “Author Keywords” and “Fractional Counting” in VOSviewer. A minimum criterion of five occurrences was established, resulting in the identification of 164 out of the total cumulative count of 1,688 terms. Afterward, the 164 keywords were refined by removing infrequent and repeated phrases such as “alga,” “animal,” “biofuels,” “biological oxygen demand analysis,” “fatty acids,” “effluent” and “biomass productions,” among others. Consequently, a total of 97 keywords were selected (see Supplementary Table S5) and represented in Figure 3. The sizes of the vertices correspond to the frequency of occurrence of the keywords. Examples of bigger vertices in the graph are “wastewater treatment,” “wastewater,” “dairy wastewater,” and “microalgae,” indicating a greater frequency of occurrence. Moreover, it can be inferred that a major number of studies were conducted on dairy wastewater treatment using microalgae and biomass production. Moreover, the colors of the vertices distinguish the clusters; that is, every term is categorized into a unique group based on its distance from the other keywords. For example, the terms “dairy wastewater treatment,” “chemical oxygen demand,” “growth rate,” and “phycoremediation,” are represented by a single color, highlighting their significant interdependence. Furthermore, there might be a notable association between words that are part of other groups, such as “mixotrophy,” dairy wastewater,” “fermentation,” and “biofuel.” In many studies, DW was used as a nutrient source for microalgae growth and further, the biomass used for biofuel production (Chokshi et al., 2016; Singh et al., 2023; Ravi Kiran et al., 2024). Singh et al. (2023) showed the potential of *Monoraphidium* sp. KMC4 biomass generated from DW towards bio-oil production. In another study, 29.6% of bio-oil yield was obtained from microalgae *Messastrum gracile* SVMIICT7 grown on dairy wastewater (Ravi Kiran et al., 2024). Three major species, namely, *Chlorella*, *Scenedesmus,* and *Acutodesmus* were reported to be superior microalgae in DW treatment (Chokshi et al., 2016; Daneshvar et al., 2019). Based on the grouping of keywords, the research on dairy wastewater treatment using microalgae may be categorized into three groups: chemical oxygen demand, biochemical composition, and biomass production.

### 4.3 Mechanisms by which microalgae can treat wastewater

Microalgae cultivation and biomass production with the integration of wastewater treatment has garnered significant interest in the recent few years. Furthermore, the economic viability of the underlying methodology and operational procedures continues to pose challenges. Several investigations were conducted on the screening of potential microalgal strains (Mohanty and Mohanty, 2023a; Singh et al., 2023), the optimization of culture conditions (Divya Kuravi and Venkata Mohan, 2021; Kiran and Venkata Mohan, 2021; 2022; Singh et al., 2023), the design of bioreactors for microalgal cultures (Arora et al., 2021), and other factors to increase the lipid content of microalgae and promote their growth. To reduce overall costs, many studies were conducted on coupling microalgae growth with dairy wastewater treatment (Singh et al., 2023; Ravi Kiran et al., 2024). The inorganic and organic nutrients present in the wastewater can be utilized by microalgae (da Silva et al., 2021). Hence, this research topic has great potential and will be helpful in the development of a novel environmentally friendly method that combines both the production of microalgae and wastewater treatment (Table 5).

**TABLE 5:** Literature covering studies conducted on dairy wastewater treatment using microalgae.

Microalgal species	Conditions	Biomass concentration or productivity	Potential product	Remarks	References
*Chlorella variabilis Scenedesmus obliquus*	Diluted synthetic DW	673 mg L^1^	Lutein	The consortia exhibited phosphate removal of 70.19%, ammoniacal nitrogen removal of 86.22%, COD removal of 54.72%.	Gatamaneni Loganathan et al. (2020)
*C. minutissima N. muscorum Spirulina* sp.	70% DW + 10 g/L of glucose at 18:6 h photoperiod	5.76 ± 0.06 g/L	Biodiesel feedstock	Polyculture removed about 58.76% of nitrogen. Removal of phosphate was observed in the range of 83%–84%.	Chandra et al. (2021)
*Ascochloris* sp. ADW007	Indoor bench-scale in DW	0.102 ± 0.003 g/L/d	Biodiesel	Over 95.1% of COD was reduced in both indoor and outdoor cultivation	Kumar et al. (2019b)
*Ascochloris* sp. ADW007	Outdoor pilot-scale in DW	0.207 ± 0.003 g/L/d	Biodiesel	Over 95.1% of COD was reduced in both indoor and outdoor cultivation	Kumar et al. (2019b)
*Chlorella* sp.	5% DW outdoor	47.50 mg L^1^ day^1^	Biodiesel	Total COD removal efficiency%: 21.99 ± 0.66 Total N removal efficiency%: 85.12 ± 3.37 Total P removal efficiency%: 88.47 ± 3.90	Lu et al. (2015)
*Chlorella* sp.	10% DWW outdoor	160.0 mg L^1^ day^1^	Biodiesel	Total COD removal efficiency%: 38.88 ± 4.09 Total N removal efficiency%: 85.17 ± 0.35 Total P removal efficiency%: 66.68 ± 1.29	Lu et al. (2015)
*Chlorella* sp.	25% DWW outdoor	110.0 mg L^1^ day^1^	Biodiesel	Total COD removal efficiency%: 54.82 ± 0.91 Total N removal efficiency%: 83.83 ± 1.19 Total P removal efficiency%: 65.33 ± 2.05	Lu et al. (2015)
*Chlorella* sp./*C. zofingiensis*	Autoclaved DW	5.41 g L^1^	Biodiesel	The highest COD removal of appx 50% was achieved.	Qin et al. (2016)
*Scenedesmus* spp*./C. zofingiensis*	Autoclaved DW	5.11 g L^1^	Biodiesel	The highest COD removal of 62.87% was achieved.	Qin et al. (2016)
*A. dimorphus*	DW	840.67 ± 11.11 mg/L	Biodiesel and Bioethanol	Maximum removal of COD, i.e. 91.71% was observed. Total (100%) removal of nitrite and ammoniacal nitrogen was observed.	Chokshi et al. (2016)
*Monoraphidium* sp.	Synthetic DW	50 mg L^1^ d^1^	Biofertilizers and nutraceutical	COD, nitrates, and phosphates removal efficiencies were 75%, 85%, and 60% respectively.	Divya Kuravi and Venkata Mohan (2022)
*Tetradesmus* sp. SVMIICT4	Synthetic DW	2.38 g L^1^	Biodiesel	The removal efficiency of 95.5% COD was observed. Nitrates/phosphates removal efficiency of 65.2/57.35% was observed.	Kiran and Venkata Mohan (2022)
*Monoraphidium* sp. KMC4	Simulated Synthetic DW	3.69 g L^1^	Bio-oil	The COD removal efficiency of 93.4% was achieved in 12.5% SSDW while the lowest (73.95%) was observed in 100% SSDW.	Singh et al. (2023)
*Monoraphidium* sp. KMC4	Synthetic DW	1.9 g L^1^	Wide spectrum biopesticide	The reduction efficiency was reported to be 96% when the initial COD of the medium was 500 mg/L, it is reduced to 78% when the initial COD of the medium was maintained at 2000 mg/L.	Mohanty and Mohanty (2023a)

#### 4.3.1 Heterotrophic mode

In heterotrophic mode, the microalgae use the respiration process to obtain energy by organic compound oxidation (Kim et al., 2019). Glucose, glycerol, and acetate are the main forms of carbon used for the cultivation of microalgae in heterotrophic mode. The use of glucose as an organic carbon source for microalgae culture has been widespread due to its superior energy in terms of adenosine triphosphate compared to other substrates. Acetate is also a common utilizable carbon source for growing microalgae in heterotrophic mode. Upon entry into the cytoplasm of microalgae cells, the process of acetate metabolism occurs via the acetylation of coenzyme A by acetyl-CoA synthetase. This reaction is a single-step process that utilizes a solitary ATP molecule, resulting in the formation of acetyl coenzyme A (acetyl-CoA). Two major pathways, namely, the glyoxylate cycle and Tricarboxylic Acid Cycle (TCA) further degrade the acetate to malate and citrate, respectively. Nevertheless, large amounts of acetate may exhibit toxicity against several cells, hence impeding their structure (da Silva et al., 2021). Also, bacteria coexisting with microalgae were found to improve the degradation of nitrogen, phosphate, glucose, and chemical oxygen demand but with a trade-off in lipid productivity (Zhang et al., 2012). *Chlorella* sp. HS2 high-density algal cultures were produced in heterotrophic cultivation mode using BG11 media with glucose in a fermenter with dark conditions. An increase of the model to a 5-L fermenter revealed that the culture depleted the phosphorus completely, which led to insufficient utilization of the nitrogen and carbon sources (Kim et al., 2019). The respiration of organic-C during heterotrophic cultivation by microalgae generates CO_2_, which contributes to the greenhouse effect. On the other hand, the coexistence of heterotrophic and autotrophic microalgae in mixed cultures may result in a reduction of carbon dioxide emissions. This is due to the mutually beneficial nutrient requirements of each microorganism, whereby the heterotrophic species consume oxygen and generate carbon dioxide. The high quantities of organic compounds in the growth medium used for cultivating heterotrophic microalgae provide the possibility of invasion by competing bacteria and fungus, which may compromise the quality of the process and products. Heterotrophic growth of microalgae thus demands sterilization of media which can incur energy costs ranging from 20% to 30% of the overall costs of the production process. This expense might be recouped if the heterotrophic microalgae produce products with high market value (da Silva et al., 2021). For the generation of high-market-value products, there is a requirement for scale-up technology, namely, a raceway pond for microalgae cultivation. It is very difficult to sterilize a huge amount of cultivation media for large ponds in heterotrophic mode. Also, the risk of bacterial and fungal contamination will increase in such open reactors (Singh et al., 2023). From our knowledge, there are no industrial plants that use heterotrophic mode of cultivation to treat DW. However, additional investigation is required to augment biomass productivity and the productivity of high-value-added compounds when DW is used as a nutrient source to overcome the high market value.

#### 4.3.2 Mixotrophic mode

In comparison to the heterotrophic mode, mixotrophic cultivation facilitates a higher growth rate and biomass productivity. To produce biochemical compounds and accomplish maximum biomass productivity, a balance between photosynthesis and respiration is important (Singh et al., 2023). The utilization of microalgae biomass as a source of renewable energy and its interconnection with numerous biological processes for the production of value-products for their subsequent reuse in a closed-loop biorefinery system facilitates many advantages and makes the process both sustainable and economically feasible (Divya Kuravi and Venkata Mohan, 2022). Many studies have provided evidence of the proliferation of microalgae, lipid synthesis, and the production of high-value products using dairy wastewater (DW) as a nutrient source in a mixotrophic mode. *Monoraphidium* sp. SVMIICT6 was identified and cultured using a mixotrophic approach to treat synthetic dairy effluent. The growth of microalgae was facilitated by the removal of nutrients, as evidenced by the carbohydrate, protein, and lipid content (25%), in addition to biomass productivity of 0.05 g L^−1^day^−1^. From PSII to PSI, both the quantum yield and the electron transport rate (ETR) enhanced throughout time, and this rise was strongly correlated with chlorophyll pigments. Heptadecanoic acid and myristoleic acid were found as significant fatty acids which has numerous nutraceutical benefits (Divya Kuravi and Venkata Mohan, 2022). Another species of *Monoraphidium* genera, *Monoraphidium* sp. KMC4 reported significant biomass production together with significant removal of pollutants from simulated synthetic dairy wastewater. This species also showed a good lipid profile and demonstrated its potential as feedstock for bio-oil (Singh et al., 2023). Also, poly-culture was reported to produce better biomass yield compared to mono-culture in raw DW (RDW). Also, the addition of cyanobacteria in polyculture assimilates nitrogen at a better rate compared to control. It is noteworthy that the biomass yield of poly-microalgae cultures CNSS (*Chlorella minutissima* + *Nostoc muscorum* + *Spirulina* sp.) and SNSS (*Scenedesmus abundans* + *Nostoc muscorum* + *Spirulina* sp.) was relatively greater than that of polymicroalgae culture CS (*C. minutissima* + *Scenedesmus abundans*). Also, biomass and lipid productivity were greater in poly-microalgae cultures. This phenomenon could be attributed to the fact that strains belonging to the same group may have competed for substrates from the cultivation medium to generate energy for their metabolic activities, resulting in a reduced biomass yield compared to poly-microalgae cultures comprising strains from two distinct groups (Chandra et al., 2021). The microalgae cultivation in outdoor open culture using RDW was also compared with indoor cultivation. The highest biomass production in indoor bench-scale cultures reached 0.26 g L^−1^ day^−1^, whereas outdoor conditions only achieved 0.11 g L^−1^ day^−1^. Also, saturated fatty acids, i. e., C16:0/C18:0 were dominant acids in outdoor biomass which indicates huge potential for cultivation of *Chlorella* sp. in RDW for high-quality biodiesel production with the trade-off in fatty acid methyl ester productivity compared to indoor cultivation (Lu et al., 2015). Contrastingly, in another outdoor cultivation of *Ascochloris* sp. ADW007 in RDW, the biomass productivity was higher (0.207 ± 0.003 g/L/d) than in the indoor bench scale study (0.102 ± 0.003 g/L/d) (Kumar et al., 2019b). In many studies, consortia of microalgae/cyanobacteria and bacteria were used to treat dairy wastewater. One of the primary benefits of microalgae consortia in wastewater treatment is their ability to enhance resilience and compensate for the loss of individual algal species during culture. The consortium consisting of *Chlorella* sp. and *C. zofingiensis* had the highest biomass concentration and productivity, with values of 5.41 g L^−1^ and 773.2 mg L^−1^ day^−1^, respectively. The growth of *Chlorella* sp. alone resulted in the highest total lipid content (21.09%) but the consortium (*Scenedesmus* spp./*C. zofingiensis*) exhibited the best lipid productivity (150.6 mg L^−1^ day^−1^) (Qin et al., 2016). Hence, the selection of microalgal consortia will depend on the final product requirement. The mixotrophic condition is not restricted to inorganic carbon only and sunlight because of the availability of organic carbon present in dairy wastewater. Still, microbial contamination is a major bottleneck in the case of mixotrophic cultivation. To overcome this drawback, a strategy to use extremophilic algae which could tolerate the inhibition and toxicity of high ammonium nitrogen and urea in dairy wastewater. *Chlorella vulgaris* CA1, isolated from dairy effluent, exhibited a remarkable tolerance to a significant concentration of ammonia nitrogen (2.7 g/L), surpassing the tolerance of other *Chlorella* species by more than 20 times. The resilience of the algae to withstand a significant concentration of ammonium nitrogen indicates the possibility of efficiently recycling nutrients from dairy effluent, while simultaneously generating algal biomass and valuable bioproducts (Pang et al., 2020). It is also important to study pigment fluorescence and photosystem transients to estimate the photosynthetic efficiency of microalgae during DW treatment. The growth of *Tetradesmus* sp. SVMIICT4 is accompanied with a reduction in nutrients in wastewater and an improvement in photosystems electron transport and pigment biosynthesis in synthetic DW. The increase in chlorophyll content (18.94 mg g^−1^) was shown to be correlated with a greater absorption flux per reaction centre, increases electron transport and decreases non-photochemical quenching. In mixotrophic mode, the process of *de-novo* fatty acid synthesis occurs in the stroma of chloroplasts, followed by the assimilation of fatty acids from acyl Co-A into the glycerol backbone. This is followed by acyl transfers, resulting in the production of unsaturated fatty acids (55.55%) and saturated fatty acids (54.42%) (Kiran and Venkata Mohan, 2022). The incorporation of biological methods into wastewater treatment within a biorefinery framework entails the creation of bio-based products that tackle environmental issues with remediation.

##### 4.3.2.1 Influence of bacteria on cultivation and wastewater treatment

The bacteria-microalgae symbiotic association in wastewater treatment is complex and can have inhibitory and stimulatory effects. Due to the absence of sterile conditions in wastewater systems, the naturally existing bacterial consortium can dominate during the cultivation of microalgae. The presence of a consortium is influenced by factors such as the composition of the wastewater, conditions, reactor design, and operational circumstances (Mathew et al., 2022). Bacteria and microalgae often engage in competition for the same nutrients within their surrounding ecosystem. When there is a scarcity of resources like nitrogen, phosphate, and carbon, bacteria have the potential to surpass microalgae in competition, resulting in a decrease in microalgal proliferation. However, the bacteria facilitate the proliferation of microalgae by supplying CO_2_, phytohormones, remineralized macro, and micronutrients. Microalgae, in turn, facilitate the growth of bacteria by providing O_2_ and organic compounds (Talapatra et al., 2023). Based on the circumstances of the growth conditions, a “natural” equilibrium is achieved between microalgae and bacteria. Nevertheless, the constitution of the consortia in this state of balance might vary significantly according to the existing circumstances inside the reactor. The composition of the consortium has a direct impact on the proportions of several phenomena, such as oxygen generation, CO_2_ consumption, nitrogen, and phosphorus assimilation. Consequently, the levels of these processes fluctuate in accordance with the changes in consortia dynamics (Mathew et al., 2022). Furthermore, microalgae may use inorganic carbon, nitrogen, and phosphorus that are generated as a result of bacterial metabolism. In many studies, the synergistic link between algae and bacteria has been shown to significantly improve the efficiency of nutrient removal. In addition to eliminating nutrients, the algal-bacterial consortium also has the ability to eliminate micropollutants, heavy metals, and pharmaceutical compounds. The mutual exchange of CO_2_ and O_2_ between algae and bacteria results in a significant reduction in costs due to the *in-situ* production of oxygen via photosynthesis by microalgae. Researchers have reported that nutrient or contaminant removal in the algal-bacteria consortium is superior in comparison to algal and conventional systems due to multiple pathways available via algal-bacterial symbiotic relations. Nitrogen is depleted due to nitrification-denitrification metabolism along with ammonium stripping when pH rises above 9. And, phosphorous gets assimilated into biomass through phosphorylation via a biological mechanism. The phosphorus gets precipitated at pH levels above and similar to 9. Despite owning several benefits, the competitive interaction and inhibitory mechanisms present in algal-bacterial systems are unclear (Oruganti et al., 2022).

Certain bacteria synthesize products that can impede the development of microalgae. These chemicals consist of antibiotics, volatile organic compounds, or secondary metabolites that have a detrimental effect on microalgae. Another major hindrance is the availability of bacteria in wastewater which can form biofilm. These biofilms can obstruct the passage of light and the absorption of nutrients by microalgae, therefore impeding their growth. Also, bacteria can alter the pH, redox potential, or oxygen concentrations in the environment. For example, elevated rates of bacterial respiration may lead to a reduction in oxygen levels, resulting in anaerobic circumstances that are unfavorable for the growth of microalgae (Mathew et al., 2022). However, selecting the inoculum size or ratio (microalgae to bacteria) can influence the overall microalgal biomass productivity and treatment efficiency. Many investigations have been reported by researchers on the effect of microalgae to bacteria/activated sludge ratio on wastewater treatment efficiency. Amini et al. examined the inoculum ratio of algae to activated sludge for domestic wastewater treatment. It was noted that the algae: sludge inoculum ratio of 5:1 compared with 1:1 and 1:5, has exhibited the highest levels of ammonium and phosphorus removal efficiency. This suggested that high inoculum levels of microalgae exhibit better results (Amini et al., 2020). In a separate investigation, Kim et al. (2014) demonstrated that the presence of *Rhizobium* sp. in co-culture with *Chlorella Vulgaris* resulted in a 72% increase in cell count. This enhancement was attributed to the mutualistic interaction between the two organisms. Also, the biomass-settling properties of algal-bacterial cultures are enhanced by the formation of granules or aggregates. The downstream processing was facilitated by the extracellular polymeric substance formation, which was attributed to the mutual interaction between bacteria and microalgae (Mathew et al., 2022). Another major concern during mixotrophic cultivation is parasitism, which can negatively harm the microalgae growth. Many bacteria produce enzymes like cellulases which can lyse the cell wall of microalgae, lead to the utilization of intracellular compounds of microalgae, and inhibit microalgal productivity (Fuentes et al., 2016). Also, the nutrient competition results in the slow growth rate of particular strains and ultimately outperforms their existence after many growth cycles (Ramanan et al., 2016). In one study by Zhang et al. (2012), the *Chlorella pyrenoidosa* impeded the growth of bacteria under high carbon concentrations. Still, the mechanism of the consortium is unclear which represents mutualism, commensalism, and parasitism mechanism. A cell-to-cell signaling known as a quorum sensing (QS) system between bacteria and microalgae is important in response to better wastewater treatment efficiency and biomass productivity. Many bacteria secrete indole acetic acid, N-acyl-homoserine lactones, and auto-inducing peptides, which act as signaling molecules in a reactor system. In one study by Amin et al., indole-3-acetic acid secreted by *Sulfitobacter* bacteria enhanced the proliferation or cell division in diatoms (Amin et al., 2015). According to Das et al., incorporating quorum-sensing molecules obtained from anaerobic sludge into the *Chlorella Sorokiniana* culture resulted in a 2.25-fold increase in algal production and a 1.8-fold rise in lipid content. The bacterial QS compounds were determined to be bacterial siderophores, autoinducing oligopeptides, N-Hexanoyl-L-homoserine lactone, and N-3-oxohexanocyl-L-homoserine lactone. The research also found that the algal cells released chemicals that disrupt quorum sensing (QS), such as β cyclodextrin, dimethyl sulphohonio propionate, 5-4-5-bromomethylene-3-butyl-2-5 H-furanone, and halogenated furanones, which deactivate bacterial toxins. Microalgae have self-protective reactions when faced with environmental constraints, such as bacterial competition (Das et al., 2019). The QS molecules produced by wastewater-born microbial consortiums (activated sludge) enhanced the lipid productivity in *Chlorophyta* sp. culture and an insignificant reduction of biomass production was observed (Zhang et al., 2018). In another study, *Azospirillum brasilense* secreted indole-3-acetic acid had promoted *C. sorokiniana* growth but at the expense of energy reserves such as neutral lipids and starch (Peng et al., 2020). Unfortunately, there are still additional gaps in comprehending these interactions between algae and bacteria. There is a significant need to investigate the sensing processes between algae and bacteria, since this research may aid in establishing effective solutions for large-scale systems.

##### 4.3.2.2 Mitigation strategies for enhancing microalgae cultivation in dairy wastewater: Addressing bacterial interference

Based on the above discussion it can be concluded that the presence of diverse bacterial communities in dairy wastewater poses a considerable challenge to the cultivation of microalgae as a competition for nutrients, produce inhibitory substances, and alter the overall microbial ecosystem. To enhance the efficiency and reliability of microalgae cultivation in this environment, various mitigation strategies can be implemented.

Pre-treatment processes are essential for reducing the bacterial load in dairy wastewater before it is introduced to microalgae cultivation systems. One effective pre-treatment method is physical filtration, which removes larger particles and a portion of the bacterial content, thereby decreasing nutrient competition. Additionally, UV irradiation is a non-chemical method that can significantly reduce microbial populations by damaging bacterial DNA. This approach is advantageous as it avoids introducing residual chemicals into the system. Chemical disinfection, using agents like chlorine or ozone, can also be effective in reducing bacterial counts. However, careful control is necessary to prevent residual chemicals from negatively impacting microalgae (Passero et al., 2014; Qin et al., 2014).

Selecting microalgae strains that are naturally resistant to bacterial inhibition or that can coexist harmoniously with specific bacterial communities is another effective strategy. Strain screening involves identifying and using strains that have demonstrated resilience in mixed microbial environments (Pintado et al., 2023). These strains can maintain high productivity even in the presence of potentially inhibitory bacteria. Additionally, genetic engineering techniques can be employed to develop microalgae strains with enhanced resistance to bacterial metabolites or other stress factors, thereby improving their suitability for cultivation in dairy wastewater.

Maintaining optimal environmental conditions can significantly influence the balance between microalgae and bacterial growth. Key factors to control include light intensity and photoperiod, pH, temperature, and nutrient management (Andrade et al., 2021). Optimizing light conditions can enhance algal photosynthesis while inhibiting bacterial proliferation, as bacteria often have different light requirements (Maltsev et al., 2021). Similarly, adjusting pH and temperature to levels optimal for microalgae but less favorable for bacteria can help reduce microbial competition (Beltrán-Rocha et al., 2024). Fine-tuning the nutrient composition and concentration can support algal growth while limiting bacterial overgrowth, ensuring that microalgae have a competitive advantage.

In some instances, the use of selective antimicrobial agents can help control bacterial populations without harming microalgae. Algal-produced antimicrobials, which are compounds naturally secreted by certain microalgae strains, can be particularly effective in inhibiting specific bacterial groups. Additionally, the careful use of selective antibiotics can target harmful bacteria while minimizing impacts on microalgae. It is crucial, however, to ensure that the use of antimicrobial agents does not lead to resistance development or negatively affect the overall microbial ecosystem (Mohanty and Mohanty, 2023b; 2023a).

Thus, addressing the challenges posed by bacterial interference in microalgae cultivation in dairy wastewater requires a multifaceted approach. By implementing a combination of pre-treatment processes, co-cultivation techniques, selective strain use, controlled environmental conditions, and the use of antimicrobial agents, it is possible to create a more favorable environment for microalgae growth. These mitigation strategies not only enhance the efficiency and productivity of microalgae cultivation but also contribute to the sustainability and feasibility of using dairy wastewater as a valuable resource for biofuel production and bioremediation. Continued research and optimization of these strategies will further improve the robustness and scalability of microalgae cultivation systems in wastewater environments.

### 4.4 Nutrient removal capabilities of microalgae

#### 4.4.1 Removal of N, P, and COD

The organic matter present in dairy wastewater is the major contaminant that need to be treated in any wastewater treatment method (Vieira Costa et al., 2021). The ability of microalgae to treat DW has been studied by several researchers. COD quantifies the concentration of organic molecules in the DW. The COD of dairy effluent decreased by more than 90% (2,593.33 ± 277.37 to 215 ± 7.07 mg/L) using *Acutodesmus dimorphus* following cultivation for 4 days. The observed reduction in COD indicates that microalgal cells possess the ability to effectively use an organic form of carbon as a building block for their metabolism. Extending the treatment time did not have a substantial impact on decreasing the COD level (Chokshi et al., 2016). Kuravi and Venkata Mohan reported a maximum removal efficiency of 75.5% of the organic content from synthetic dairy wastewater by microalgae contributing to its growth and photosynthetic activity (Divya Kuravi and Venkata Mohan, 2022). Acetate undergoes metabolism via the glyoxylate route to produce malate, which serves as a precursor for the production of fatty acids (da Silva et al., 2021). On the other hand, algae convert carbon dioxide into organic matter by harnessing ATP and NADPH via the Calvin cycle (Mohsenpour et al., 2021). Microalgae use carbon dioxide as their primary source during photoautotrophic mode. The dissociation of gaseous CO_2_ into bicarbonate and carbonate ions in water is dependent upon the pH level. The specific equilibrium between these ions is influenced by factors such as temperature, cations amount, and salinity. The carbon dioxide can simply pass through the plasma membrane of cells due to the non-polar nature of gas while the bicarbonate requires an active transport system. Through the enzymatic activity of carbonic anhydrase, bicarbonate is quickly catalyzed to CO_2_ in the chloroplast, promoting the fixation of inorganic carbon. The majority of microalgae have developed carbon concentration mechanisms to mitigate the decline in photosynthetic performance, hence enhancing the rate of carbon dioxide accumulation. This adaptation is mostly driven by the low CO_2_ concentration in water. The Calvin cycle converts an inorganic form of carbon to an organic form of carbon via CO_2_ fixing to the acceptor molecule (Ribulose-1,5-bisphosphate) in the presence of RuBisCo (Ribulose-1,5-bisphosphate carboxylase oxygenase) enzyme to yield 2 molecules of 3-phosphoglycerate and is subsequently forming Glyceraldehyde-3-phosphate. During this process, the production of four molecules of Ribulose-1,5-bisphosphate occurs for every three molecules of carbon dioxide that are fixed, leaving just three molecules left in the cycle. The one molecule of Glyceraldehyde-3-phosphate is either stored or further converted into pyruvate and then into the tricarboxylic acid cycle (Mohsenpour et al., 2021). Prior research has shown that the quality of light has a significant role in determining the rate at which microalgae grows. The induction of high photosynthetic machinery is attributed to the high absorption of photosystems I and II for red and blue wavelengths, respectively. The impact of light wavelengths on the production and productivity of microalgal biomass in airy wastewater was found to be significant. Under cool-white fluorescent light, the highest yield of 673 mg L^−1^ was reported. The protein content in microalgae was highest under cool-white fluorescent light. In contrast, amber light increased carbohydrate content, whereas red light increased lipid composition. Cool fluorescent illumination outperforms other wavelengths because the photosynthetic rate is increased when the number of light-harvesting antennas increases and when the chlorophyll receives light at 600–700 nm (Gatamaneni Loganathan et al., 2020).

Also, microalgae are capable of removing significant nitrogen and phosphate. Nitrogen is available in the form of ammonium, which can be toxic to microalgae. Therefore, strains that are tolerant to high concentrations of ammonium should be used to treat such effluent. Nitrogen is supplied as an important source of growth for microalgae during cultivation. Nitrate is used as a supplement in synthetic culture medium while ammonium form of nitrogen is present in effluent for microalgae (Vieira Costa et al., 2021). According to reports, ammoniacal nitrogen is the preferred nitrogen source for microalgae due to its direct metabolism and low energy requirements for absorption (Singh et al., 2023). After 6 days of cultivation, all ammoniacal nitrogen was consumed by *A. dimorphus* from RDW (277.4 ± 10.75 mg/L) (Chokshi et al., 2016). Kuravi and Venkata Mohan reported that the nitrogen was reduced from 165 mg L^−1^–27.1 mg L^−1^ using *Monoraphidium* sp. in dairy wastewater, revealing a maximum treatment efficiency of 83.5% (Divya Kuravi and Venkata Mohan, 2022). Singh et al. observed a significant reduction of ammonium-N in 50% simulated synthetic dairy wastewater (SSDW) using *Monoraphidium* sp. KMC4, resulting in a removal efficiency of 90.56%. Contrastingly, the lower removal efficiency was attained in 100% and 75% SSDW respectively. This can be explained that a high amount of ammonium-N in wastewater might have inhibited the viability and hampered the metabolism of cells (Singh et al., 2023). A prior investigation has also shown that an elevated concentration of ammonium-N has the potential to impede the proliferation of microalgae (Lin et al., 2021). In the mechanism of nitrogen absorption, the cellular uptake of nitrate occurs, followed by its reduction to nitrite by the action of the cytosolic-NADH-dependent nitrate reductase enzyme. Following this, the nitrite is sent to the chloroplast, and it undergoes reduction to ammonium by the catalytic action of NADPH-linked nitrite reductase (Mohsenpour et al., 2021). Hemalatha et al. (2019) documented a nitrate removal efficiency of 65.5% through the cultivation of mixed microalgae in DW. In contrast, Kothari et al. (2012) observed a 90% nitrate removal from 75% DW using *Chlamydomonas polypyrenoideum* after 10 days. Phosphate is also found as an important nutrient for microalgae growth. The phosphate is found in the form of phosphate and inorganic salts in wastewater (Vieira Costa et al., 2021). In the investigation conducted by Singh et al., a removal efficiency of 84.13% was observed in 50% SSDW (Singh et al., 2023). According to Kothari et al. (2012), *C. polypyrenoideum* demonstrated a phosphate removal efficiency of 70% from 75% DW. The assimilation of phosphorus by microalgae has been classified as a component that limits their growth. The polyphosphate reserves are used to store surplus phosphorous for synthesizing phosphatides, proteins, and nucleic acids. Furthermore, phosphorous may facilitate the augmentation of cellular division and the production of ATP (Singh et al., 2023). The *Chlorella* genera has shown promising results in removing the nutrients efficiently for its growth from dairy wastewater in several studies (Mohanty and Mohanty, 2023b; Maleki Samani and Mansouri, 2023). The biomass produced in effluent holds a promising avenue toward biofuel and livestock feed production. As seen through the removal efficiency of microalgae towards contaminants from wastewater, the DW industry has the potential to prevail over the high expense of biomass production. It is recommended to conduct the process of dairy wastewater treatment along with biomass production using toxicity-tolerant microalgal strains in a biorefinery approach.

#### 4.4.2 Removal of heavy metals

**FIGURE 4:**
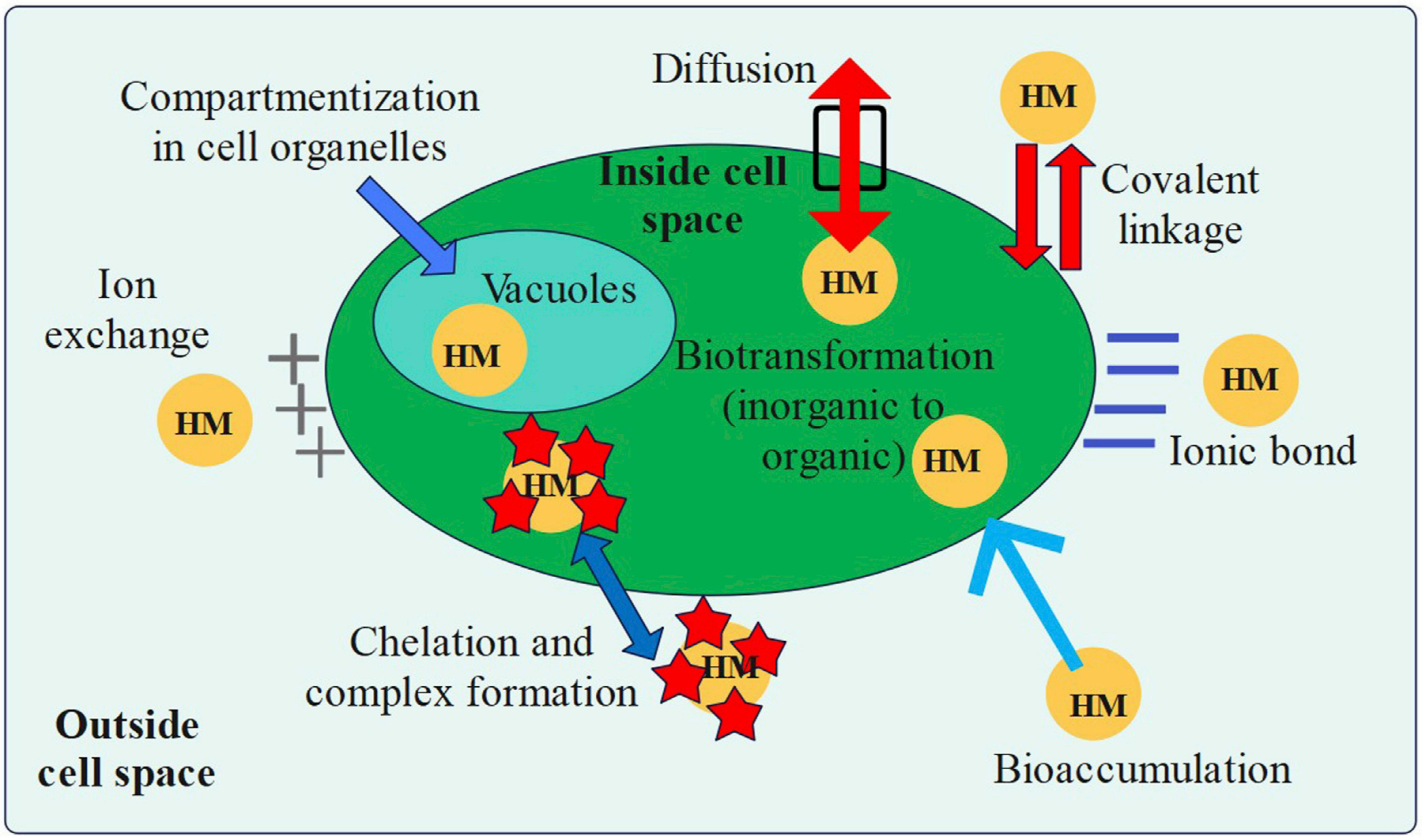
Different mechanisms for removal of heavy metals by microalgae.

An efficient, economical, and ecologically beneficial method of removing metal ions from wastewater is to employ algae for the biosorption process. However, to obtain the appropriate level of treatment with algal-based systems, it is important to have an understanding of the physiological characteristics of algae. At the microscale level, several different methods of heavy metal biosorption by algae are described. These mechanisms include ion exchange, complex formation, and electrostatic interaction. Many metals, including molybdenum, copper, zinc, nickel, manganese, iron, cobalt, and boron, are regarded to be micronutrients for cells. The trace elements play a crucial role in promoting the growth of cells by serving as essential components for cellular metabolism (Figure 4). Conversely, heavy metals such as silver, gold, aluminum, Mercury, titanium, cadmium, lead, and arsenic are detrimental to the growth of microalgae and are classified as toxic heavy metals. Microalgae are widely recognized as highly effective remediators due to their remarkable tolerance capacity, ease of cultivation, strong binding affinity, higher surface area, and the ability to utilize dead biomass (Priya et al., 2022). The microbe employs various mechanisms to protect itself from heavy metal exposure, such as gene regulation and chelation (Chugh et al., 2022). The microalgae used two methods for heavy adsorption, bio-binding or bio-removal. The initial stage involves the adsorption of heavy metals onto the surface of the cell, on the different functional groups on their cell surface. The mechanism may or may not involve metabolism in cells. The metals binding to the surface of the cell occurs through the electrostatic forces of attraction and complexation. The process is classified as passive because of the non-requirement of any form of energy. In a study conducted by Buayam et al., the experimental findings indicate that *Desmodesmus* was able to achieve a copper removal efficiency of 80%. The efficiency of Cu removal was observed to decrease at pH 4 compared to 6, suggesting that pH has an impact on the ability to remove Cu. In addition, the presence of Cu had a negative impact on the growth of algae and resulted in alterations to their ultrastructure (Buayam et al., 2019). Both living and non-living biomass can be involved in biosorption (Abdel-Raouf et al., 2012). The bioremediation of chromium was observed in the dead cells of *Phaeodactylum tricornutum* and *Navicula pelliculosa*, with an efficiency ranging from 24% to 32%. However, the efficiency of chromium in the presence of extracellular polymeric substances covering the cells ranges from 27% to 37% (Hedayatkhah et al., 2018). The binding process of metals to functional groups including sulphate, carboxyl, amino, and hydroxyl due to the presence of polysaccharides, lipids, and proteins causes flocs formation, which effectively reduces the concentration of metals. During this second phase, the method involves the movement of heavy metals through the cell’s membrane to either the cytoplasm or other organelles. The process of accumulating heavy metals inside algal cells is referred to as bioaccumulation (Abdel-Raouf et al., 2012). The accumulation process is an active process because it requires energy. The accumulation remedial process can only be performed by living cells because it is based on metabolic activity. Following the process of bioaccumulation, these pollutants undergo the process of detoxification removal or generate a harmless complex through the mechanisms of detoxification, compartmentalization, or complexation (Chugh et al., 2022). A study conducted by Wei et al. has reported that synthetic organic pollutants have been found to enhance the removal efficiency of heavy metals. The findings from the study indicate that exposure to Cr(VI) or o-nitrophenol resulted in a reduction in photosynthetic and superoxide dismutase activities of *Chlamydomonas reinhardtii*, while simultaneously leading to an increase in the generation of reactive oxygen species and malondialdehyde content. The rates of elimination of chromium (VI) and organic nitrogenous pollutants (ONP) by *C. reinhardtii* cells exhibited a substantial rise, ranging from 37.4% to 54.9% and from 35.8% to 45.9%, respectively (Wei et al., 2020). This strategy of microbial based remediation of heavy metals helps elimination of contaminants from wastewater along with reduction in chemical oxygen demand and biomass production for various applications.

### 4.5 Different types of cultivation systems applicable for dairy wastewater treatment

**FIGURE 5:**
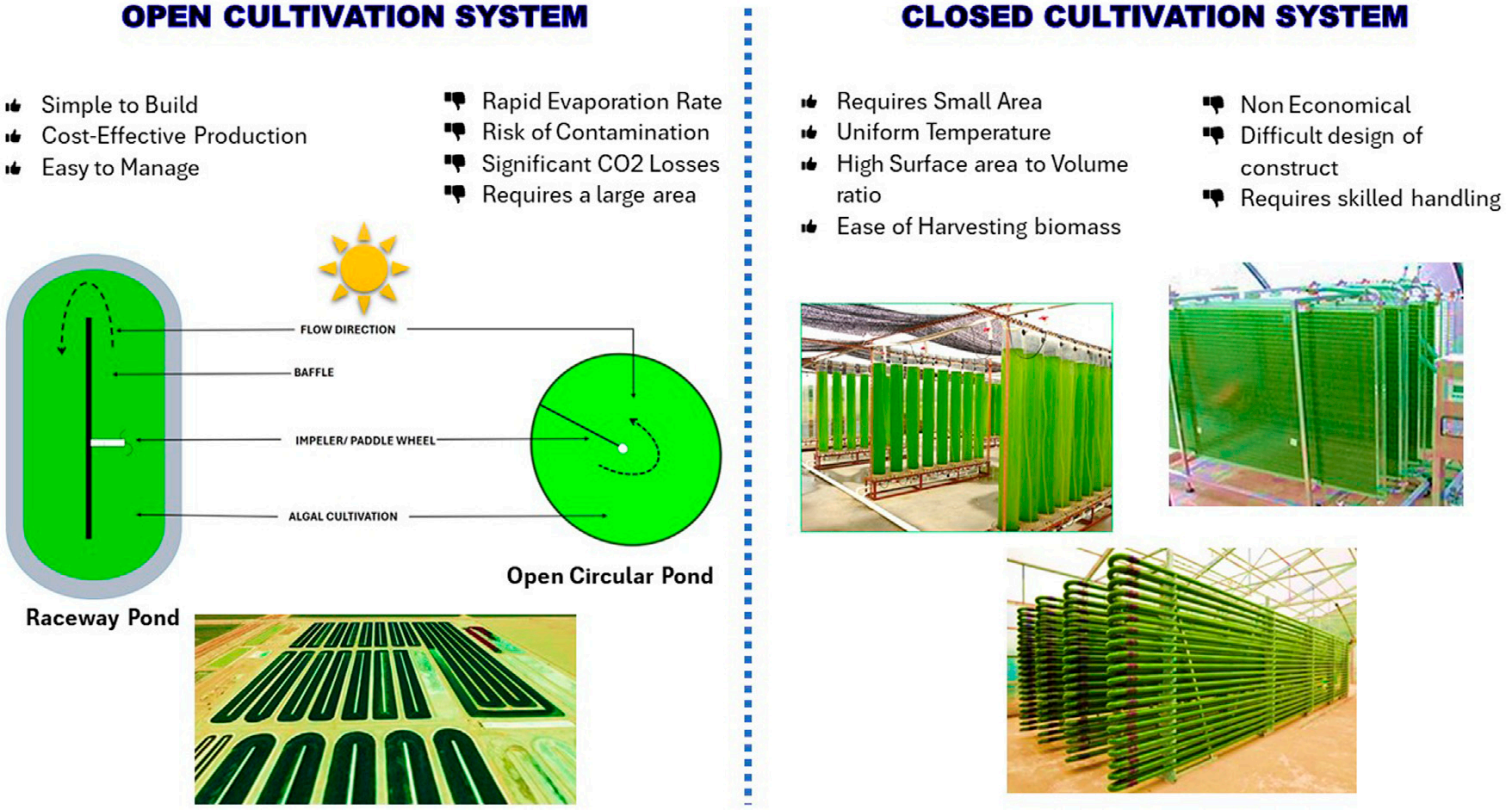
Open and closed pond system for algae cultivation.

In this part, we aim to explore the different modes of cultivation systems applicable to dairy wastewater treatment using microalgae. We will examine the principles behind each cultivation system, their advantages, challenges, and recent advancements. Additionally, we will discuss key research findings and case studies to provide insights into the performance and applicability of these systems in real-world dairy wastewater treatment scenarios. Microalgae cultivation can be conducted through both open and closed systems (Figure 5). Open systems, naturally occurring in environments like ponds, lagoons, seas, and oceans, provide a habitat for microalgae growth. Conversely, closed systems such as photobioreactors offer controlled conditions of temperature, pH, and nutrient availability to optimize biomass yield. Microalgae may be grown in unconventional sources, such as industrial effluents, which is interesting since it makes them more useful for wastewater bioremediation (Posadas et al., 2017).

#### 4.5.1 Open systems

Open ponds represent the most simplest and convenient method for large-scale microalgae cultivation. They encompass natural bodies of water like lakes and ponds, as well as human-made structures such as circular and raceway systems. In this method, shallow ponds or raceways are utilized as the cultivation environment for microalgae, harnessing the nutrient-rich nature of dairy wastewater to promote algal growth. The process begins by introducing dairy wastewater into the open ponds, providing essential nutrients such as nitrogen and phosphorus required for microalgae growth (Arora et al., 2021). Under natural sunlight, microalgae photosynthesize and utilize these nutrients, effectively removing pollutants from the wastewater. This bioremediation process helps in reducing the organic load, nitrogen, and phosphorus content in the wastewater, thus mitigating environmental pollution. In raceway ponds, mixing is typically facilitated by paddle wheels, while in circular ponds, rotating arms serve a similar purpose. Additionally, in larger ponds, mixing can be achieved in specific areas using impeller blades (Yen et al., 2019). These mixing mechanisms play a crucial role in maintaining homogeneity within the pond environment, ensuring adequate nutrient distribution, and promoting optimal conditions for microalgae growth.

Open pond cultivation presents a viable and sustainable solution for treating dairy wastewater, offering economic, operational, and environmental advantages compared to other treatment methods. Its simplicity, scalability, and efficiency make it an attractive option for dairy facilities seeking cost-effective and environmentally friendly wastewater management solutions. Open pond systems are typically less expensive to construct and operate compared to closed systems such as photobioreactors (Xiaogang et al., 2022). They require minimal infrastructure and maintenance, making them a cost-effective option for dairy wastewater treatment. Open ponds can be easily scaled up or down to accommodate varying wastewater volumes (Gupta et al., 2019). This scalability makes them suitable for both small-scale dairy operations and large-scale industrial facilities. Open ponds utilize natural sunlight for photosynthesis, eliminating the need for artificial lighting. This reduces energy consumption and operational costs associated with providing light in closed systems. Dairy wastewater is rich in nutrients such as nitrogen and phosphorus, which are essential for microalgae growth. Open pond systems leverage these nutrients, promoting robust algal biomass production. Open pond cultivation is relatively simple and straightforward, requiring minimal technical expertise. Operators can easily monitor and manage the system without sophisticated equipment or complex control systems. Open ponds can be adapted to cultivate various species of microalgae, offering flexibility in biomass production.

However, open systems come with certain limitations. They are susceptible to contamination by various microorganisms, including protozoa and bacteria, present in the surrounding environment (Lam et al., 2018). These contaminants can compete with the desired microorganisms for nutrients and space, affecting the overall productivity and purity of the culture. Unlike closed or controlled systems, open systems lack precise control over growth parameters such as temperature, pH, light intensity, and nutrient availability (Faried et al., 2017). As a result, fluctuations in environmental conditions can occur, leading to inconsistent growth and productivity of the microorganisms. Open systems are exposed to environmental factors such as weather conditions, seasonal changes, and fluctuations in water quality. These external factors can negatively impact the stability and reliability of the cultivation process, making it challenging to maintain optimal growth conditions. Scaling up open systems for large-scale production can be impractical due to space limitations and the need for extensive infrastructure (Tan et al., 2018). Additionally, achieving uniform mixing and distribution of nutrients in large open systems can be difficult, leading to uneven growth and productivity across the system. Open systems are susceptible to pest infestations and predation by insects, birds, and other wildlife (Vieira Costa et al., 2021). These pests can damage the microorganism culture or consume the biomass, resulting in reduced yields and economic losses. The discharge of effluents from open systems into natural water bodies can have environmental consequences, such as nutrient runoff and eutrophication, which can disrupt aquatic ecosystems and degrade water quality.

#### 4.5.2 Closed system (photo-bioreactors)

Given the limitations associated with pond systems, there’s a common preference to cultivate algae strains in photobioreactors. These systems allow for precise control and monitoring of operating conditions and nutrient levels through automated control systems, significantly reducing the risk of contamination (Suparmaniam et al., 2019). An ideal PBR model should incorporate the following features: 1) efficient light-harvesting capabilities to facilitate the transport, channeling, and distribution of light among microalgal species for optimal biomass production; 2) the ability to maintain operational parameters feasibly to promote high utilization of light energy by the cells; 3) minimized investment and operational costs; and 4) reduced energy consumption (Xiaogang et al., 2022). Two prevalent types of photobioreactors include straight tubes, which are either arranged horizontally on the ground or vertically in long rows called tubular bioreactors, and helical bioreactors, consisting of spirally wound tubes around a central support. These bioreactors commonly employ tubes made of glass or perpex. Tubular bioreactors are predominantly utilized outdoors and can be oriented vertically, horizontally, inclined, or helically to optimize sunlight exposure, thereby enhancing photosynthesis and maximizing algal biomass production (Ting et al., 2017). Photobioreactors (PBRs) should be designed to be straightforward, cost-effective, and capable of achieving high volumetric productivity while remaining energy-efficient and suitable for scaling up to industrial levels. Tubular bioreactors exhibit a specific limitation in their photosynthetic efficiency, resulting in higher energy consumption. A significant drawback of these photobioreactors is the uneven concentration gradient along the lengthy tubes, leading to inadequate mass transfer (Tan et al., 2021). Furthermore, the growth of cells in the central region is hampered by reduced photosynthesis due to oxygen toxicity, which can manifest within just 1 minute in a tube lacking proper gas exchange (Arora et al., 2021). Additionally, closed systems like tubular bioreactors are prone to uncontrolled proliferation of pathogenic microorganisms on inner surfaces, forming biofilms that impede reagent mass transfer due to external resistance at the biofilm interface (Skoneczny and Tabiś, 2015). Plastic bag photobioreactors are gaining popularity for their cost-effectiveness and varying volumes, typically constructed from polythene (Wang et al., 2012). However, challenges arise from difficulties in mixing components and the bags’ susceptibility to damage, potentially reducing the system’s longevity. Despite their benefits, closed systems incur high operational and construction expenses.

One major hurdle associated with photobioreactors (PBRs) in microalgal biomass production lies in the substantial expenses incurred in their construction and maintenance. While these high costs may render PBRs impractical for biodiesel production, they hold promise for producing high-value compounds with greater commercial potential. Researchers such as Nugroho and Zhu have suggested strategies to mitigate operational expenses, including the utilization of cost-effective materials like wastewater as a feedstock and the adoption of energy-efficient pumps for resource recovery (Nugroho and Zhu, 2019). Another significant challenge faced by PBRs is the gradual limitation of light penetration on the surface where algal biofilms develop. However, advancements in bioengineered PBR designs offer solutions to operational issues while maintaining high efficiency and minimizing maintenance costs. For instance, Wu et al. (2019) developed an innovative algal biofilm photobioreactor using hog manure wastewater, resulting in significant *C. vulgaris* growth and easy harvesting via a scraping method. Additionally, in response to the light attenuation issue arising from suspended solids and contaminants in anaerobically digested wastewater (ADW), Chen et al. implemented a hollow fiber membrane (HFM) system within the photobioreactor. This setup enables nutrients to permeate from the inner chamber containing ADW to the outer chamber housing the algal culture medium via the HFM. Consequently, this configuration effectively controls pollutants, mitigating the inhibition caused by suspended particles (Chen et al., 2018). One more significant disadvantage of using photobioreactors for treating dairy industry wastewater is the potential for fouling and clogging. Dairy wastewater contains organic compounds, nutrients, and suspended solids, which can accumulate and form biofilms on the surfaces of the photobioreactor, obstructing light penetration and inhibiting algal growth. This fouling can decrease the efficiency of the photobioreactor, leading to reduced wastewater treatment performance and increased maintenance requirements. Additionally, the presence of fats, oils, and proteins in dairy wastewater may further exacerbate fouling issues, requiring frequent cleaning and maintenance to prevent system failure. Therefore, managing fouling and clogging challenges is a crucial consideration when implementing photobioreactors for dairy wastewater treatment.

#### 4.5.3 Case studies

Several researchers have investigated microalgae’s ability to remove nutrients from dairy effluent. For example, Huo et al. (2012) explored the outdoor cultivation of *Chlorella zofingiensis* and its effectiveness in nutrient removal from dairy effluent. They compared the impact of two pH regulation methods, 6% CO_2_ and acetic acid, on the removal rates of total nitrogen (TN) and orthophosphate. Their findings showed that after 6 days of cultivation, the use of CO_2_ resulted in higher removal rates for TN (51.7%) and orthophosphate (97.5%) compared to acetic acid (TN = 79.6%; orthophosphate = 42.0%) for pH control. Guruvaiah et al. (2015) conducted a study to evaluate the potential of *Chloromonas playfairii* and *Desmodesmus opoliensis* for nutrient removal from dairy effluent. Both strains achieved over 90% removal of COD, ammonium-N, and total phosphorus. After 15 days of cultivation, maximum biomass concentrations reached 1.7 g L^−1^ and 1.2 g L^−1^, with corresponding maximum lipid concentrations of 15% and 12%. In a separate study, Lu et al. (2015) investigated *Chlorella* sp.’s nutrient removal capability from DW in indoor and outdoor cultures. Results indicated significant differences in nutrient removal rates between the two conditions. Indoor cultures showed notably higher removal rates for COD, total nitrogen (N), and phosphorus (P) compared to outdoor cultures. Specifically, indoor conditions exhibited removal rates of 88.38 mg L^−1^ d^−1^ for COD, 38.34 mg L^−1^ d^−1^ for total N, and 2.03 mg L^−1^ d^−1^ for P, while outdoor conditions showed rates of 41.31 mg L^−1^ d^−1^ for COD, 6.58 mg L^−1^ d^−1^ for total N, and 2.74 mg L^−1^ d^−1^ for P. Moreover, indoor cultures demonstrated higher maximum biomass productivity, with levels of 260 mg L^−1^ d^−1^ compared to 110 mg L^−1^ d^−1^ in outdoor cultures when cultivated in dairy wastewater. Pandey et al. (2019) conducted a study to assess the efficiency of effluent treatment and lipid accumulation by cultivating the microalgae *Scenedesmus* sp. ASK22 in DW. The findings demonstrated promising results for both effluent treatment and lipid productivity. The study reported significant removal efficiencies for various pollutants present in the dairy effluent, including 100% removal for nitrate, 98.63% removal for phosphorus, and over 99% removal for chemical oxygen demand (COD). These high removal efficiencies underscore the effectiveness of *Scenedesmus* sp. ASK22 in treating DW, thereby reducing pollutant levels and enhancing effluent quality. Additionally, the study observed a lipid productivity of 31.16 mg L^−1^ d^−1^, indicating the potential of *Scenedesmus* sp. ASK22 for lipid accumulation. Given the interest in microalgal lipids for biodiesel production and other value-added products, the observed lipid productivity suggests that *Scenedesmus* sp. ASK22 holds promise as a candidate for lipid production using dairy effluent as a growth medium.

The above studies collectively illustrate the potential of microalgae-based treatment systems in remediating dairy effluent and sustainably producing valuable bioproducts. These findings underscore microalgae’s promising role in addressing dairy effluent challenges and suggest further research to optimize cultivation strategies and explore additional applications in wastewater treatment and biorefinery sectors. Various cultivation conditions, including indoor and outdoor cultures and different bioreactor setups, were explored to optimize biomass productivity and pollutant removal. Microalgae-based treatment effectively reduced pollutants like COD, nitrate, and phosphate, demonstrating their eco-friendly wastewater treatment potential.

### 4.6 Potential for biomass production and value-added products from microalgae cultivated in dairy wastewater

**FIGURE 6:**
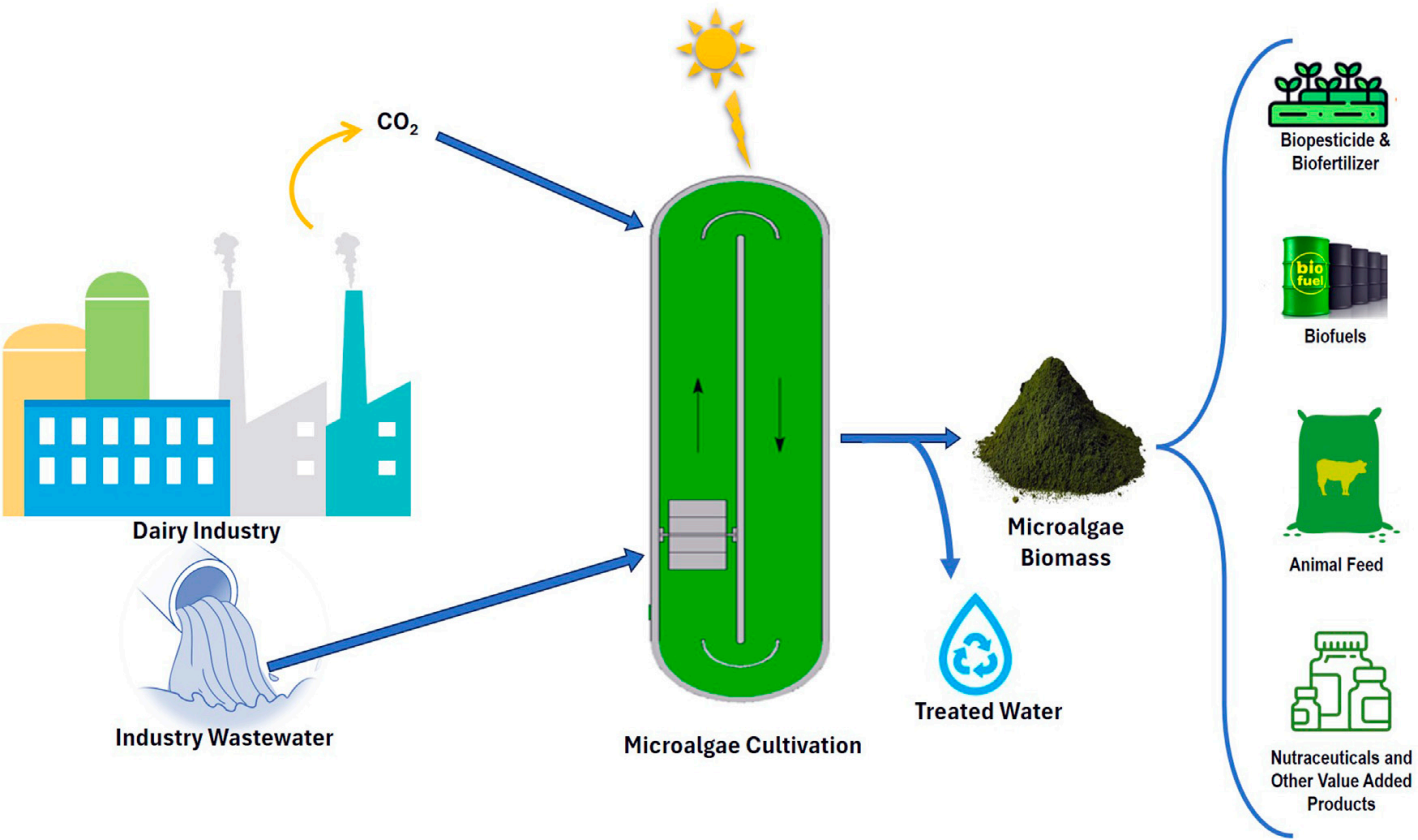
Applications of microalgae biomass grown in effluents.

Microalgae are renowned for their capacity to generate bioactive substances, encompassing antibiotics, vaccines, antibodies, hepatotoxic and neurotoxic agents, hormones, enzymes, and various therapeutic compounds (Rizwan et al., 2018). Additionally, the pigments found in microalgae have exhibited potential health benefits, including the prevention of cancer, mitigation of heart disease, support for neurological health, and prevention of eye diseases. Notably, microalgae possess advantageous traits such as rapid growth and the ability to thrive in uncomplicated, cost-effective growth media, rendering them optimal hosts for synthesizing recombinant proteins. Moreover, their post-translational modifications closely mirror those of mammalian cells, surpassing bacterial cells in this regard (Khavari et al., 2021). Microalgae biomass produced from effluents is not directly suitable for human consumption but finds applications in energy, animal feed, and agriculture (Acién Fernández et al., 2018). However, its utilization is hindered by the high biomass quantity and production costs (Costa et al., 2019b). Nutrient recovery from wastewater for microalgae production could enhance biomass availability for applications like fertilizer or biofuel production (Figure 6).

#### 4.6.1 Biofuel

Microalgae biomass derived from dairy effluents holds promise for energy applications, offering a CO_2_-neutral alternative to fossil fuels (Pandey et al., 2019; Kumar et al., 2020; Shahid et al., 2020). These microorganisms demonstrate versatility in nutrition modes, encompassing autotrophic, heterotrophic, and mixotrophic modes, thereby enhancing their suitability for biofuel production (Pandey et al., 2019). While biodiesel production from microalgae shows potential, its large-scale implementation faces challenges such as biomass generation with high productivity, lipid content, extraction methods, and water usage (Yin et al., 2020). Pyrolysis presents an alternative approach for biofuel generation, yielding bio-oil, biochar, and biogas without biomass residue (Li. et al., 2019). Fast pyrolysis is favored for microalgae, ensuring high bio-oil yields by minimizing secondary reactions through rapid heating rates and short residence times (Bridgwater, 2012). Notably, the growth of *Nostoc ellipsosporum* in municipal wastewater has been investigated to optimize biomass production, nutrient removal efficiency, and bio-oil yields. Various formulations of municipal wastewater as growth media enabled biomass yield enhancement from 1.42 to 2.9 g L^−1^ post optimization and acclimation. The process achieved notable nitrogen and phosphate removal efficiencies of 87.59% and 88.31%, respectively, alongside a bio-oil yield of 24.62% at 300°C (Devi and Parthiban, 2020). Microalgae species with high carbohydrate content (>40%) like *Chlamydomonas, Spirulina, Euglena, Chlorella, Scenedesmus,* and *Dunaliella* have been investigated for bioethanol production (Mehar et al., 2019).

#### 4.6.2 Biofertilizer

The escalating global food demand and environmental contamination from extensive chemical fertilizer usage underscore the significance of biostimulants, biopesticides, and biofertilizers (Plaza et al., 2018; Costa et al., 2019a; Castro et al., 2020; Kour et al., 2020). In sustainable agriculture, biofertilizers are increasingly recognized for enhancing vegetable crop productivity in eco-friendly and economically feasible manners, mitigating the adverse impacts of synthetic fertilizers. Among biofertilizers, those derived from photosynthetic organisms like microalgae are gaining prominence for their significant contributions to soil fertility and crop yield enhancement. Biofertilizers offer a favorable substitute for chemical fertilizers due to their lower toxicity and minimal side effects (Li et al., 2017). *Chlorella* stands out as one of the extensively studied microalgae genera worldwide, particularly notable for its wide usage in agricultural applications and wastewater treatment due to its robust nutrient removal capabilities (Garrido-Cardenas et al., 2018; Li et al., 2019). The research evaluated the impact of both fresh and dry *C. vulgaris* biomass as a biofertilizer on lettuce seedling growth, observing significant enhancements in seedling parameters and pigment content compared to unfertilized plants (Vieira Costa et al., 2021). Similarly, Uysal et al. (2015), demonstrated the efficacy of *C. vulgaris* as an agricultural biofertilizer, reporting improved seed germination rates and enhanced growth in wheat and corn plants when treated with liquid-cultivated microalgae under autotrophic conditions. Another promising candidate for agricultural use is *Spirulina*, which has been employed since 1981 as a substitute for chemical fertilizers and for soil restoration (Vieira Costa et al., 2021). Plaza et al. (2018), investigated the effects of foliar spraying with *S. platensis* and *Scenedesmus* sp. on the development of Petunia x hybrida plants and leaf nutrient status. The study demonstrated that foliar application of *Spirulina* led to increased root dry matter, flower count per plant, and water content. Conversely, the application of *Scenedesmus* accelerated root growth, leaf and shoot development, and early flowering. Additionally, the study highlighted the potential of microalgae hydrolysate in enhancing plant nutritional status. Microalgae offer multiple benefits in organic agriculture, serving as a safe nitrogen source without causing pollution or toxicity to plants or consumers (Manjunath et al., 2016). Additionally, they synthesize biopesticidal metabolites, aiding in pest control (Costa et al., 2019a). Moreover, microalgae contribute to soil recovery, agricultural wastewater treatment, and heavy metal removal from soil (Abdel-Raouf N, 2012).

#### 4.6.3 Pigments

Microalgae biomass grown in wastewater can be utilized for pigment production, offering valuable compounds like chlorophylls, carotenoids, and phycobiliproteins (Daneshvar et al., 2019; Arashiro et al., 2020). Daneshvar et al., explored mixotrophic cultivation of *Scenedesmus quadricauda* and *T. suecica* in dairy industry effluent (DWW). Chlorophyll content significantly increased in both microalgae during the first cycle of mixotrophic cultivation with DWW, reaching 19.00 mg/g and 22.00 mg/g for *T. suecica* and *S. quadricauda*, respectively. Carotenoid content was also notable, with values of 6.90 mg/g for *T. suecica* and 7.76 mg/g for *S. quadricauda*. However, carotenoid concentrations decreased in the second cultivation cycle with dairy wastewater recycling, indicating potential pollutant removal efficiency. This suggests that reusing dairy wastewater in consecutive cultivation cycles can enhance pollutant removal and biomass production efficiency. Ribeiro et al. (2017), investigated the use of ricotta cheese byproduct (scotta) for *Chlorella protothecoides* cultivation, enhancing carotenoid synthesis through stress induction. This led to significant carotenoid production, including astaxanthin and lutein/zeaxanthin accumulation. Arashiro et al. (2020), studied *Nostoc* sp.*, Arthrospira platensis*, and *Porphyridium purpureum* cultivation in food industry effluents, achieving efficient pollutant removal and high-value phycobiliprotein extraction, highlighting microalgae’s potential for industrial wastewater treatment and phycobiliprotein production. These organisms demonstrated remarkable efficiency in removing up to 98% of COD, 94% of inorganic nitrogen, and 100% of phosphate. Additionally, successful extraction of phycocyanin, allophycocyanin, and phycoerythrin from the biomass yielded concentrations of 103 mg/g, 57 mg/g, and 30 mg/g dry weight, respectively.

#### 4.6.4 Animal feed

Microalgae biomass derived from dairy effluent holds the potential for animal feed production (Labbé et al., 2017). Incorporating microalgae into animal feed improves animal health and enhances the quality of animal products like meat and eggs (Yaakob et al., 2014). However, the high cost and limited availability of microalgae hinder widespread adoption. If costs decrease and availability increases, microalgae biomass could be initially integrated into the feed of young animals, with broader implementation later on. One approach to reducing production costs involves maximizing microalgae utilization, including its concurrent use in biofuels. This strategy would utilize wastewater nutrients for biomass production, which could then be used for lipid extraction for biodiesel and subsequently for producing protein-rich animal feed (Gatrell et al., 2014). Microalgae biomass surpasses traditional animal feed sources like corn, grasses, and small fish in terms of nutritional content, including proteins, essential fatty acids, and carotenoids. Additionally, microalgae contain antioxidant and antimicrobial compounds vital for disease prevention and potentially extending animals’ life cycles (Dineshbabu et al., 2019). Commonly utilized microalgae species in aquaculture include marine strains like *Nannochloropsis* and freshwater strains such as *Chlorella, Spirulina*, and *Scenedesmus* (Vieira Costa et al., 2021).

## 5 Challenges in microalgal-based treatment processes and future research directions

An integrated algae system has two primary challenges: large-scale algae production and collecting algae for downstream processing into biofuels and other valuable bioproducts. Large-scale algae cultivation has issues in nutrient supply and recycling, gas transfer and exchange, light intensity, depth, culture age, land and water availability, and harvesting. Downstream processing, accounting for 40% of overall cost, is the key challenge owing to the inability to recover numerous microalgal a biological refinery products simultaneously. Recent research suggests algae might be an option for automotive fuels. Thus, microalgae cultivation has gained popularity due to its economic value as a feedstock (Ramírez Mérida and Rodríguez Padrón, 2023). There has been a growing interest in the utilization of microalgae for wastewater treatment in recent years. Using microalgae biomass for CO_2_ fixation helps preserve the carbon footprint. This creates a self-sustaining process that benefits the environment, industry, and global life. The right reactor together with its configuration is crucial for biomass to reach and absorb the maximum amount of substrate, ensuring good yields and productivity. Also, it is important to conduct industrial-scale research that will allow to understand microalgae behaviour under high-volume settings. Moreover, finding resilient strains that are well-suited to the particular level of pollutants that provide higher yields and productivity for particular bio-products is important. Lastly, there is a need to investigate studies on the reuse of culture media in photobioreactors to increase the efficiency of the overall process. Thus, the integration of the microalgal biorefinery with the wastewater treatment concept may have significant prospects for ecological sustainability. The conversion of contaminants included in wastewater is an inevitable process that aids in environmental improvement by stabilizing the compounds before their release into aquatic environments.

## 6 Conclusion

Biological wastewater treatment using microalgae benefits the technology economically and environmentally. It may deliver effective and inexpensive tertiary treatments that minimize nitrogen, phosphorus, and chemical oxygen demand levels. Simultaneously, microalgae can fix CO_2_ which helps in the reduction of emissions of greenhouse gases and the maintenance of carbon footprints. The utilization of microalgae has garnered significant interest in the past few years, owing to the noteworthy outcomes observed in microalgae biomass at the agri-food-fuel level. This not only offers commercial benefits but also facilitates the development of sustainable development processes by generating value-added products from biomass that can be applied in a variety of sectors. Notwithstanding this, the microalgal process encounters challenges pertaining to the design of reactors or culture systems, physicochemical control variables, scaling, and microalgae harvesting. The discipline of the microalgae-based process presents substantial prospects for improving the efficiency of dairy wastewater treatment and nutrient utilization in a biorefinery model. Consequently, there is still a need to conduct research on the large-scale cultivation of microalgae in addition to promoting awareness regarding the societal benefits associated with microalgae utilization.

## Figures and Tables

**Table:** 

**DW**	Dairy wastewater
**COD**	Chemical oxygen demand
**BOD**	Biochemical oxygen demand
**FAO**	Food and Agriculture Organisation-
**PHA**	Polyhydroxyalkanoates
**PHB**	Polyhydroxybutyrate
**TSS**	Total suspended solids
**NMR**	Nuclear Magnetic Resonance
**EPA**	Exopolysaccharides
**Acetyl-CoA**	Acetyl coenzyme A
**ATP**	Adenosine triphosphate
**TCA**	Tricarboxylic Acid Cycle
**HRAP**	High-rate algal ponds
**ETR**	Electron transport rate
**RDW**	Raw dairy wastewater
**NADPH**	Nicotinamide adenine dinucleotide phosphate hydrogen
**Rubisco**	Ribulose-1,5-bisphosphate carboxylase oxygenase
**SSDW**	Simulated synthetic dairy wastewater
**ONP**	Organic nitrogenous pollutants
**PBR**	Photobioreactors
**ADW**	Anaerobically digested wastewater
**HFM**	Hollow fiber membrane
**BG-11**	Blue-green Medium
**TAP**	Tris-Acetate-Phosphate
**UF**	Ultrafiltration
**NF**	Nanofiltration
**RO**	Reverse Osmosis
**MF**	Microfiltration
**SBR**	Sequencing batch reactor
**ASBR**	Anaerobic sequencing batch reactor
**AD**	Anaerobic digestion
**QS**	Quorum sensing
**UASBRs**	Up-flow anaerobic sludge blanket reactors
**HRT**	Hydraulic retention time
**SRT**	Sludge retention time
