# Supplementary material for: Comprehensive assessment of microalgal-based treatment processes for dairy wastewater
Source: Front Bioeng Biotechnol. 2024 Aug 6;12:1425933. doi: 10.3389/fbioe.2024.1425933 (PMC11333367; doi:10.3389/fbioe.2024.1425933)
Supplement: Supplementary file 1 [file Table1.docx]

Table S1. The publication of number of documents on the treatment of dairy wastewater using microalgae

| Year | No. of articles |
| --- | --- |
| 2024 (till the date data retrieved from scopus) | 4 |
| 2023 | 24 |
| 2022 | 27 |
| 2021 | 13 |
| 2020 | 14 |
| 2019 | 12 |
| 2018 | 7 |
| 2017 | 4 |
| 2016 | 6 |
| 2015 | 3 |
| 2014 | 7 |
| 2013 | 0 |
| 2012 | 1 |
| 2011 | 1 |
| 2010 | 1 |
| 2009 | 0 |
| 2008 | 1 |
| 2007 | 0 |
| 2006 | 0 |
| 2005 | 0 |
| 2004 | 0 |
| 2003 | 0 |
| 2002 | 0 |
| 2001 | 0 |
| 2000 | 0 |
| 1999 | 0 |
| 1998 | 0 |
| 1997 | 1 |
| 1996 | 1 |

Table S2 Involvement of countries in the area of dairy wastewater treatment using microalgae

| Country | No. of articles |
| --- | --- |
| India | 36 |
| China | 18 |
| United States | 13 |
| Brazil | 10 |
| Iran | 10 |
| Greece | 9 |
| Italy | 8 |
| Taiwan | 8 |
| Australia | 6 |
| Hong Kong | 6 |
| Spain | 5 |
| Canada | 4 |
| Finland | 4 |
| Poland | 4 |
| Romania | 4 |
| Malaysia | 3 |
| Mexico | 3 |
| Portugal | 3 |
| South Africa | 3 |
| South Korea | 3 |
| Colombia | 2 |
| Qatar | 2 |
| Sweden | 2 |
| Argentina | 1 |
| Austria | 1 |
| Chile | 1 |
| Ecuador | 1 |
| Egypt | 1 |
| Ireland | 1 |
| Japan | 1 |
| Philippines | 1 |
| Russian Federation | 1 |
| Saudi Arabia | 1 |
| Singapore | 1 |
| Sri Lanka | 1 |
| Switzerland | 1 |
| Thailand | 1 |
| Uruguay | 1 |
| Viet Nam | 1 |

Table S3 Number of articles published by Universities/ Institutes till date in the area of dairy wastewater treatment using microalgae

| University | No. of articles |
| --- | --- |
| Guangzhou Institute of Energy Conversion of the Chinese Academy of Sciences | 10 |
| Chinese Academy of Sciences | 10 |
| National Cheng Kung University | 7 |
| Tunghai University | 7 |
| City University of Hong Kong | 6 |
| Indian Institute of Chemical Technology | 5 |
| University of Chinese Academy of Sciences | 5 |
| University of Patras | 5 |
| Indian Institute of Technology Guwahati | 4 |
| Yuan Ze University | 4 |
| Uniwersytet Warminsko-Mazurski w Olsztynie | 4 |
| Bialystok University of Technology | 3 |
| Universidade Federal de Vicosa | 3 |
| University of the Aegean | 3 |
| Indian Institute of Technology Roorkee | 3 |
| Indian Institute of Technology Delhi | 3 |
| Laboratório Nacional de Energia e Geologia | 3 |
| Academy of Scientific and Innovative Research AcSIR | 3 |
| School of Industrial Technology | 3 |
| Universidade Federal do Rio Grande | 2 |
| Motilal Nehru National Institute of Technology Allahabad | 2 |
| Universitatea din Bucuresti | 2 |
| New Mexico State University | 2 |
| Université McGill | 2 |
| University Politehnica of Bucharest | 2 |
| Instituto Superior Técnico | 2 |
| The University of Newcastle, Australia | 2 |
| Vellore Institute of Technology | 2 |
| Institutul National de Cercetare - Dezvoltare pentru Chimie si Petrochimie | 2 |
| Guangxi Normal University | 2 |
| Gorgan University of Agricultural Sciences and Natural Resources | 2 |
| Shanghai Jiao Tong University | 2 |
| Tamil Nadu Agricultural University | 2 |
| Universidade Federal de Itajubá | 2 |
| Sant'Anna Scuola Universitaria Superiore Pisa | 2 |
| Isfahan University of Technology | 2 |
| Daneshgahe Shahid Bahonar-e-Kerman | 2 |
| Sapienza Università di Roma | 2 |
| McGill University, Macdonald Campus | 2 |
| Sri Venkateswara College of Engineering, Sriperumbudur | 2 |
| Amity University | 2 |
| Babol Noshirvani University of Technology | 2 |
| Institutul National de Cercetare Dezvoltare pentru Ecologie Industriala-ECOIND | 2 |
| Itä-Suomen yliopisto | 2 |
| Universidade de Lisboa | 2 |
| New Mexico State University College of Engineering | 2 |
| IILM Academy of Higher Learning | 1 |
| Agricultural College and Research Institute | 1 |
| Centro Universitário Filadélfia UniFil | 1 |
| Shandong Provincial Engineering Center on Environmental Science and Technology | 1 |
| Sardar Patel Renewable Energy Research Institute SPRERI | 1 |
| ProCycla SPA | 1 |
| ProCycla SL | 1 |
| University Campus | 1 |
| Uremia University | 1 |
| Overseas Expertise Introduction Center for Discipline Innovation of Food Nutrition and Human Health 111 Center | 1 |
| Gurukula Kangri Deemed to be University | 1 |
| IID Gandhinagar | 1 |
| Teregroup Srl | 1 |
| Mirmadan Mohanlal Government Polytechnic | 1 |
| Ministry of Jal Shakti | 1 |
| SA | 1 |
| Arrabawn Co-Operative Society Ltd. | 1 |
| Independent Environmental Consultant | 1 |
| Universidade Federal de Juiz de Fora | 1 |
| University of Johannesburg | 1 |
| Universidade Federal Rural de Pernambuco | 1 |
| Universidade Federal do ABC | 1 |
| Ministry of Education of the People's Republic of China | 1 |
| Kyung Hee University | 1 |
| USDA Agricultural Research Service | 1 |
| Durban University of Technology | 1 |
| Eastern New Mexico University | 1 |
| Instituto Agronomico do Parana | 1 |
| University College Dublin | 1 |
| Universidade Federal de Alagoas | 1 |
| National Taiwan University | 1 |
| Nanyang Technological University | 1 |
| California State Polytechnic University, Pomona | 1 |
| University of Missouri | 1 |
| Oklahoma State University | 1 |
| University of Western Macedonia | 1 |
| Università degli Studi di Salerno | 1 |
| Vaasan Yliopisto | 1 |
| Luleå University of Technology | 1 |
| Maulana Abul Kalam Azad University of Technology | 1 |
| Universidad Veracruzana | 1 |
| Tecnológico de Monterrey | 1 |
| Universidade de São Paulo | 1 |
| Centro de Investigacion y Desarrollo En Criotecnologia de Alimentos CONICET- Universidad Nacional de la Plata- Comisión de Investigaciones Científicas de la Provincia de Buenos Aires | 1 |
| MU College of Veterinary Medicine | 1 |
| Empresa Brasileira de Pesquisa Agropecuária - Embrapa | 1 |
| Fudan University | 1 |
| Częstochowa University Of Technology | 1 |
| Universitat de Girona | 1 |
| University of KwaZulu-Natal | 1 |
| California Polytechnic State University, San Luis Obispo | 1 |
| CRS4 - Centro di Ricerca Sviluppo e Studi Superiori in Sardegna | 1 |
| Universitat Politècnica de València | 1 |
| Tanta University | 1 |
| Nagasaki University | 1 |
| Politecnico di Torino | 1 |
| Università degli Studi di Milano-Bicocca | 1 |
| Oregon State University | 1 |
| Pondicherry University | 1 |
| University of Florida | 1 |
| Sardar Patel University | 1 |
| Indian Institute of Science | 1 |
| LUT University | 1 |
| Universitatea de Stiinte Agronomice si Medicina Veterinara din Bucuresti | 1 |
| University of Trás-os-Montes and Alto Douro | 1 |
| RUDN University | 1 |
| Universidade Federal de Uberlândia | 1 |
| Sri Krishnadevaraya University | 1 |
| Università degli Studi di Pavia | 1 |
| Aristotle University of Thessaloniki | 1 |
| Middlebury College | 1 |
| Dalhousie University | 1 |
| Centro de Investigacion y de Estudios Avanzados del Instituto Politécnico Nacional | 1 |
| Jiangsu University | 1 |
| National Chung Hsing University | 1 |
| Joint Institute for High Temperatures of the Russian Academy of Sciences | 1 |
| Maejo University | 1 |
| Washington State University Pullman | 1 |
| Tripura University | 1 |
| University of Missouri School of Medicine | 1 |
| Università degli Studi dell'Aquila | 1 |
| Indian Institute of Technology BHU Varanasi | 1 |
| Instituto Politécnico Nacional | 1 |
| Foundation for Research and Technology-Hellas | 1 |
| Council of Scientific and Industrial Research India | 1 |
| International Crops Research Institute for the Semi-Arid Tropics | 1 |
| Monash University | 1 |
| University of Southern Queensland | 1 |
| Jadavpur University | 1 |
| Consiglio Nazionale delle Ricerche | 1 |
| The University of Toledo | 1 |
| Jawaharlal Nehru Technological University Hyderabad | 1 |
| Institute of Chemical Engineering Sciences | 1 |
| Universitat Autònoma de Barcelona | 1 |
| Universidad Autónoma Metropolitana - Azcapotzalco | 1 |
| Politecnico di Milano | 1 |
| USDA ARS Salinity Laboratory | 1 |
| Catholic Kwandong University | 1 |
| South China University of Technology | 1 |
| Universidad de Valladolid | 1 |
| Instituto de Ecología, A.C. | 1 |
| Central Salt & Marine Chemicals Research Institute | 1 |
| University of Michigan, Ann Arbor | 1 |
| Universität Wien | 1 |
| Shiraz University | 1 |
| Virginia Polytechnic Institute and State University | 1 |
| Sveriges lantbruksuniversitet | 1 |
| University of Seoul | 1 |
| Sharif University of Technology | 1 |
| National Taiwan University of Science and Technology | 1 |
| Universidade Federal Rural do Rio de Janeiro | 1 |
| Alma Mater Studiorum Università di Bologna | 1 |
| Università di Pisa | 1 |
| National and Kapodistrian University of Athens | 1 |

Table S4 Number of articles published and studied in the Subject area

| Subject area | No. of articles |
| --- | --- |
| Environmental Science | 92 |
| Chemical Engineering | 44 |
| Energy | 44 |
| Agricultural and Biological Sciences | 24 |
| Biochemistry, Genetics and Molecular Biology | 18 |
| Engineering | 11 |
| Chemistry | 7 |
| Immunology and Microbiology | 6 |
| Medicine | 5 |
| Social Sciences | 5 |
| Business, Management and Accounting | 2 |
| Computer Science | 2 |
| Pharmacology, Toxicology and Pharmaceutics | 2 |
| Physics and Astronomy | 1 |

Table S5 Interconnected keywords involved in search throughout this area

| Keyword | Occurrences | Total link strength |
| --- | --- | --- |
| Activated sludge | 5 | 5 |
| *Acutodesmus* *obliquus* | 6 | 6 |
| Agricultural wastes | 6 | 6 |
| Algae | 49 | 49 |
| Algal growth | 29 | 29 |
| Ammonia | 26 | 26 |
| Anaerobic digestion | 11 | 11 |
| Animals | 9 | 9 |
| Article | 56 | 56 |
| Bacteria | 13 | 13 |
| Biochemical composition | 6 | 6 |
| Biochemical oxygen demand | 17 | 17 |
| Biodiesel | 19 | 19 |
| Bioenergy | 6 | 6 |
| Bioethanol | 5 | 5 |
| Biofilm | 6 | 6 |
| Biofuel | 35 | 35 |
| Biohydrogen | 5 | 5 |
| Biomass | 71 | 71 |
| Biomass production | 33 | 33 |
| Biomass productivity | 6 | 6 |
| Bioreactor | 13 | 13 |
| Biorefinery | 5 | 5 |
| Bioremediation | 28 | 28 |
| Biosynthesis | 5 | 5 |
| Biotechnology | 8 | 8 |
| Carbohydrate | 13 | 13 |
| Carbon | 11 | 11 |
| Carbon dioxide | 8 | 8 |
| Chemical oxygen demand | 35 | 35 |
| *Chlorella* | 24 | 24 |
| Chlorophyll | 6 | 6 |
| Cultivation | 18 | 18 |
| Cyanobacteria | 9 | 9 |
| Dairy effluents | 6 | 6 |
| Dairy farming | 12 | 12 |
| Dairy industry | 15 | 15 |
| Dairy manures | 5 | 5 |
| Dairy wastewater | 58 | 56 |
| Dairy wastewater treatment | 13 | 12 |
| Digestate | 5 | 5 |
| Dry weight | 7 | 7 |
| Ecology | 6 | 6 |
| Effluent treatment | 10 | 10 |
| Effluents | 20 | 20 |
| Fatty acid | 15 | 15 |
| Feedstocks | 8 | 8 |
| Fermentation | 8 | 8 |
| Fertilizers | 6 | 6 |
| Growth rate | 13 | 13 |
| Hydrolysis | 5 | 5 |
| Illumination | 5 | 5 |
| Industrial waste | 9 | 9 |
| Light intensity | 5 | 5 |
| Lipid | 29 | 29 |
| Lipid storage | 8 | 8 |
| Manures | 8 | 8 |
| Metabolism | 17 | 17 |
| Microalgae | 83 | 83 |
| Microalgae cultivation | 6 | 6 |
| Microalgal biomass | 5 | 5 |
| Microbial community | 7 | 7 |
| Microbiology | 8 | 8 |
| Microorganisms | 42 | 42 |
| Mixotrophy | 7 | 7 |
| Municipal wastewater | 6 | 6 |
| Nitrate | 7 | 7 |
| Nitrogen | 43 | 43 |
| Nonhuman | 56 | 56 |
| Nutrients | 26 | 26 |
| Organic carbon | 6 | 6 |
| Palmitic acid | 7 | 7 |
| pH | 15 | 15 |
| Phosphate | 13 | 13 |
| Photobioreactor | 12 | 12 |
| Photosynthesis | 10 | 10 |
| Phycoremediation | 9 | 9 |
| Pollutant removal | 26 | 26 |
| Protein | 13 | 13 |
| Reclamation | 8 | 8 |
| Recycling | 5 | 5 |
| *Scenedesmus* | 14 | 14 |
| Sewage | 20 | 20 |
| Sustainable development | 7 | 7 |
| Waste treatment | 9 | 9 |
| Waste water management | 35 | 35 |
| Wastewater | 71 | 71 |
| Wastewater treatment | 84 | 80 |
| Water | 5 | 5 |
| Water management | 7 | 7 |
| Water pollutant | 7 | 7 |
| Water pollution | 5 | 5 |
| Water purification | 7 | 7 |
| Water quality | 6 | 6 |
| Water treatment | 8 | 8 |
| Yeast | 6 | 6 |
